# Supplementary material for: Different roles of concurring climate and regional land-use changes in past 40 years’ insect trends
Source: Nat Commun. 2022 Dec 12;13:7611. doi: 10.1038/s41467-022-35223-3 (PMC9744861; doi:10.1038/s41467-022-35223-3)
Supplement: Supplementary file 1 — Supplementary Information [file 41467_2022_35223_MOESM1_ESM.pdf]

Supplementary Information

Article Title: Different roles of concurring climate and regional land-use changes in past 40 years' insect trends

Authors: Felix Neff, Fränzi Korner-Nievergelt, Emmanuel Rey, Matthias Albrecht, Kurt Bollmann, Fabian Cahenzli, Yannick Chittaro, Martin M. Gossner, Carlos Martínez-Núñez, Eliane S. Meier, Christian Monnerat, Marco Moretti, Tobias Roth, Felix Herzog, Eva Knop

Corresponding Author: Felix Neff, Agroecology and Environment, Agroscope, Reckenholzstrasse 191, 8046 Zurich, Switzerland, Tel. 0041 58 463 54 20, mail@felixneff.ch

Table S1 List of species that were included in the analyses (i.e. species with sufficient data), sorted along insect group and family. Habitat specialisation (standardised per group) and temperature niche are given for all species. Critical species, which were excluded in sensitivity analyses, are indicated: migratory, (re)introduced (incl. synanthrope species), uncertain taxonomic status, difficult identification (resulting in a small amount of reliable data available and thus potentially an underrepresentation of the situation of the species in Switzerland).

Group	Family	Species	Specialisation	T. niche	Critical
Butterflies	Hesperiidae	*Carcharodus alceae*	0.70	11.4 °C	
Butterflies	Hesperiidae	*Carterocephalus palaemon*	0.20	6.7 °C	
Butterflies	Hesperiidae	*Erynnis tages*	0.50	9.0 °C	
Butterflies	Hesperiidae	*Hesperia comma*	0.50	8.3 °C	
Butterflies	Hesperiidae	*Heteropterus morpheus*	0.40	9.4 °C	
Butterflies	Hesperiidae	*Muschampia floccifera*	0.50	9.7 °C	
Butterflies	Hesperiidae	*Muschampia lavatherae*	0.90	10.7 °C	
Butterflies	Hesperiidae	*Ochlodes sylvanus*	0.10	8.4 °C	
Butterflies	Hesperiidae	*Pyrgus accreta*	0.80	8.3 °C	
Butterflies	Hesperiidae	*Pyrgus alveus*	0.30	7.3 °C	
Butterflies	Hesperiidae	*Pyrgus andromedae*	0.70	3.3 °C	
Butterflies	Hesperiidae	*Pyrgus armoricanus*	0.70	10.3 °C	
Butterflies	Hesperiidae	*Pyrgus cacaliae*	0.50	5.1 °C	
Butterflies	Hesperiidae	*Pyrgus carlinae*	0.60	6.7 °C	
Butterflies	Hesperiidae	*Pyrgus carthami*	0.70	9.6 °C	
Butterflies	Hesperiidae	*Pyrgus malvae*	0.10	8.0 °C	
Butterflies	Hesperiidae	*Pyrgus malvoides*	0.30	11.2 °C	
Butterflies	Hesperiidae	*Pyrgus onopordi*	0.90	11.7 °C	
Butterflies	Hesperiidae	*Pyrgus serratulae*	0.50	8.9 °C	
Butterflies	Hesperiidae	*Pyrgus warrenensis*	0.80	4.0 °C	difficult ID
Butterflies	Hesperiidae	*Spialia sertorius*	0.60	11.1 °C	
Butterflies	Hesperiidae	*Thymelicus acteon*	0.80	11.7 °C	
Butterflies	Hesperiidae	*Thymelicus lineola*	0.00	8.5 °C	
Butterflies	Hesperiidae	*Thymelicus sylvestris*	0.00	9.8 °C	
Butterflies	Lycaenidae	*Agriades glandon*	0.70	5.3 °C	
Butterflies	Lycaenidae	*Agriades optilete*	0.90	3.7 °C	
Butterflies	Lycaenidae	*Agriades orbitulus*	0.70	3.0 °C	
Butterflies	Lycaenidae	*Aricia agestis*	0.80	10.1 °C	
Butterflies	Lycaenidae	*Aricia artaxerxes*	0.60	5.5 °C	
Butterflies	Lycaenidae	*Aricia nicias*	0.90	2.9 °C	
Butterflies	Lycaenidae	*Cacyreus marshalli*	1.00	12.5 °C	(re)introduced
Butterflies	Lycaenidae	*Callophrys rubi*	0.50	8.2 °C	
Butterflies	Lycaenidae	*Celastrina argiolus*	0.90	8.7 °C	
Butterflies	Lycaenidae	*Cupido alcetas*	0.80	10.7 °C	
Butterflies	Lycaenidae	*Cupido argiades*	0.50	9.3 °C	
Butterflies	Lycaenidae	*Cupido minimus*	0.40	8.4 °C	
Butterflies	Lycaenidae	*Cupido osiris*	0.80	9.7 °C	
Butterflies	Lycaenidae	*Cyaniris semiargus*	0.30	7.2 °C	
Butterflies	Lycaenidae	*Eumedonia eumedon*	0.80	4.8 °C	
Butterflies	Lycaenidae	*Favonius quercus*	0.90	9.4 °C	
Butterflies	Lycaenidae	*Glaucopsyche alexis*	0.70	9.9 °C	
Butterflies	Lycaenidae	*Iolana iolas*	0.90	10.8 °C	
Butterflies	Lycaenidae	*Kretania trappi*	1.00	3.9 °C	
Butterflies	Lycaenidae	*Lampides boeticus*	0.90	12.4 °C	migratory
Butterflies	Lycaenidae	*Lycaena alciphron*	0.70	9.3 °C	
Butterflies	Lycaenidae	*Lycaena dispar*	0.90	8.8 °C	
Butterflies	Lycaenidae	*Lycaena helle*	0.70	5.4 °C	
Butterflies	Lycaenidae	*Lycaena hippothoe*	0.30	5.9 °C	
Butterflies	Lycaenidae	*Lycaena phlaeas*	0.70	9.2 °C	

Butterflies	Lycaenidae	*Lycaena tityrus*	0.20	9.5 °C	
Butterflies	Lycaenidae	*Lycaena virgaureae*	0.50	6.3 °C	
Butterflies	Lycaenidae	*Lysandra bellargus*	0.60	10.6 °C	
Butterflies	Lycaenidae	*Lysandra coridon*	0.30	9.4 °C	
Butterflies	Lycaenidae	*Phengaris alcon*	0.60	9.1 °C	
Butterflies	Lycaenidae	*Phengaris arion*	0.70	8.9 °C	
Butterflies	Lycaenidae	*Phengaris nausithous*	1.00	8.2 °C	
Butterflies	Lycaenidae	*Phengaris teleius*	1.00	8.2 °C	
Butterflies	Lycaenidae	*Plebejus argus*	0.50	7.8 °C	
Butterflies	Lycaenidae	*Plebejus argyrognomon*	0.80	9.1 °C	
Butterflies	Lycaenidae	*Plebejus idas*	0.40	5.9 °C	
Butterflies	Lycaenidae	*Polyommatus amandus*	0.80	6.7 °C	
Butterflies	Lycaenidae	*Polyommatus damon*	0.80	8.4 °C	
Butterflies	Lycaenidae	*Polyommatus daphnis*	1.00	9.5 °C	
Butterflies	Lycaenidae	*Polyommatus dorylas*	0.60	9.0 °C	
Butterflies	Lycaenidae	*Polyommatus eros*	0.80	7.1 °C	
Butterflies	Lycaenidae	*Polyommatus escheri*	0.80	10.8 °C	
Butterflies	Lycaenidae	*Polyommatus icarus*	0.20	8.7 °C	
Butterflies	Lycaenidae	*Polyommatus thersites*	0.80	10.5 °C	
Butterflies	Lycaenidae	*Pseudophilotes baton*	0.50	10.2 °C	
Butterflies	Lycaenidae	*Satyrium acaciae*	0.80	10.1 °C	
Butterflies	Lycaenidae	*Satyrium ilicis*	0.90	10.2 °C	
Butterflies	Lycaenidae	*Satyrium pruni*	0.90	8.5 °C	
Butterflies	Lycaenidae	*Satyrium spini*	0.70	10.6 °C	
Butterflies	Lycaenidae	*Satyrium w-album*	0.90	8.7 °C	
Butterflies	Lycaenidae	*Scolitantides orion*	0.90	9.4 °C	
Butterflies	Lycaenidae	*Thecla betulae*	0.90	8.5 °C	
Butterflies	Nymphalidae	*Aglais io*	0.70	8.4 °C	
Butterflies	Nymphalidae	*Aglais urticae*	0.70	7.3 °C	
Butterflies	Nymphalidae	*Apatura ilia*	0.90	9.1 °C	
Butterflies	Nymphalidae	*Apatura iris*	1.00	8.2 °C	
Butterflies	Nymphalidae	*Aphantopus hyperantus*	0.20	7.7 °C	
Butterflies	Nymphalidae	*Araschnia levana*	0.80	8.3 °C	
Butterflies	Nymphalidae	*Argynnis paphia*	0.90	8.8 °C	
Butterflies	Nymphalidae	*Boloria aquilonaris*	1.00	3.9 °C	
Butterflies	Nymphalidae	*Boloria dia*	0.80	9.3 °C	
Butterflies	Nymphalidae	*Boloria euphrosyne*	0.70	6.1 °C	
Butterflies	Nymphalidae	*Boloria napaea*	0.70	2.7 °C	
Butterflies	Nymphalidae	*Boloria pales*	0.60	6.1 °C	
Butterflies	Nymphalidae	*Boloria selene*	0.40	6.4 °C	
Butterflies	Nymphalidae	*Boloria thore*	0.80	2.2 °C	
Butterflies	Nymphalidae	*Boloria titania*	0.50	6.3 °C	
Butterflies	Nymphalidae	*Brenthis daphne*	0.70	10.0 °C	
Butterflies	Nymphalidae	*Brenthis ino*	0.60	6.4 °C	
Butterflies	Nymphalidae	*Brintesia circe*	0.90	11.2 °C	
Butterflies	Nymphalidae	*Chazara briseis*	0.90	10.5 °C	
Butterflies	Nymphalidae	*Coenonympha arcania*	0.70	9.2 °C	
Butterflies	Nymphalidae	*Coenonympha darwiniana*	0.70	4.6 °C	
Butterflies	Nymphalidae	*Coenonympha gardetta*	0.30	6.0 °C	
Butterflies	Nymphalidae	*Coenonympha glycerion*	0.90	7.9 °C	
Butterflies	Nymphalidae	*Coenonympha pamphilus*	0.20	9.1 °C	
Butterflies	Nymphalidae	*Coenonympha tullia*	0.90	5.9 °C	
Butterflies	Nymphalidae	*Erebia aethiops*	0.60	8.0 °C	
Butterflies	Nymphalidae	*Erebia albergana*	0.80	6.6 °C	
Butterflies	Nymphalidae	*Erebia arvernensis*	0.80	7.0 °C	

Butterflies	Nymphalidae	*Erebia bubastis*	0.80	3.2 °C	taxonomic status
Butterflies	Nymphalidae	*Erebia epiphron*	0.60	7.2 °C	
Butterflies	Nymphalidae	*Erebia eriphyle*	0.80	4.9 °C	
Butterflies	Nymphalidae	*Erebia euryale*	0.70	7.1 °C	
Butterflies	Nymphalidae	*Erebia flavofasciata*	0.90	2.9 °C	
Butterflies	Nymphalidae	*Erebia gorge*	0.80	6.1 °C	
Butterflies	Nymphalidae	*Erebia ligea*	0.90	4.7 °C	
Butterflies	Nymphalidae	*Erebia manto*	0.50	6.2 °C	
Butterflies	Nymphalidae	*Erebia medusa*	0.50	7.7 °C	
Butterflies	Nymphalidae	*Erebia melampus*	0.40	5.3 °C	
Butterflies	Nymphalidae	*Erebia meolans*	0.90	8.8 °C	
Butterflies	Nymphalidae	*Erebia mnestra*	0.70	3.8 °C	
Butterflies	Nymphalidae	*Erebia montana*	0.90	5.6 °C	
Butterflies	Nymphalidae	*Erebia oeme*	0.60	7.0 °C	
Butterflies	Nymphalidae	*Erebia pandrose*	0.70	2.0 °C	
Butterflies	Nymphalidae	*Erebia pharte*	0.50	5.0 °C	
Butterflies	Nymphalidae	*Erebia pluto*	0.90	4.7 °C	
Butterflies	Nymphalidae	*Erebia pronoe*	0.50	6.1 °C	
Butterflies	Nymphalidae	*Erebia styx*	1.00	5.8 °C	
Butterflies	Nymphalidae	*Erebia sudetica*	0.70	6.1 °C	
Butterflies	Nymphalidae	*Erebia triarius*	0.90	8.9 °C	
Butterflies	Nymphalidae	*Erebia tyndarus*	0.60	4.4 °C	
Butterflies	Nymphalidae	*Euphydryas aurinia*	0.40	10.0 °C	
Butterflies	Nymphalidae	*Euphydryas cynthia*	0.90	5.3 °C	
Butterflies	Nymphalidae	*Euphydryas intermedia*	0.90	4.0 °C	
Butterflies	Nymphalidae	*Fabriciana adippe*	0.50	7.6 °C	
Butterflies	Nymphalidae	*Fabriciana niobe*	0.50	7.5 °C	
Butterflies	Nymphalidae	*Hipparchia fagi*	0.70	10.6 °C	
Butterflies	Nymphalidae	*Hipparchia genava*	0.60	9.1 °C	
Butterflies	Nymphalidae	*Hipparchia semele*	0.50	9.7 °C	
Butterflies	Nymphalidae	*Hipparchia statilinus*	0.90	12.2 °C	
Butterflies	Nymphalidae	*Hyponephele lycaon*	0.70	8.9 °C	
Butterflies	Nymphalidae	*Issoria lathonia*	0.60	9.3 °C	
Butterflies	Nymphalidae	*Lasiommata maera*	0.60	8.1 °C	
Butterflies	Nymphalidae	*Lasiommata megera*	0.50	10.5 °C	
Butterflies	Nymphalidae	*Lasiommata petropolitana*	0.60	4.6 °C	
Butterflies	Nymphalidae	*Libythea celtis*	0.90	12.4 °C	
Butterflies	Nymphalidae	*Limenitis camilla*	1.00	9.1 °C	
Butterflies	Nymphalidae	*Limenitis populi*	1.00	6.6 °C	
Butterflies	Nymphalidae	*Limenitis reducta*	0.90	11.6 °C	
Butterflies	Nymphalidae	*Lopinga achine*	1.00	8.2 °C	
Butterflies	Nymphalidae	*Maniola jurtina*	0.30	9.9 °C	
Butterflies	Nymphalidae	*Melanargia galathea*	0.60	9.7 °C	
Butterflies	Nymphalidae	*Melitaea asteria*	1.00	2.1 °C	
Butterflies	Nymphalidae	*Melitaea athalia/celadussa*	0.50	7.7 °C	
Butterflies	Nymphalidae	*Melitaea aurelia*	0.70	7.8 °C	
Butterflies	Nymphalidae	*Melitaea cinxia*	0.80	9.7 °C	
Butterflies	Nymphalidae	*Melitaea deione*	0.90	11.7 °C	
Butterflies	Nymphalidae	*Melitaea diamina*	0.40	7.8 °C	
Butterflies	Nymphalidae	*Melitaea didyma*	0.70	10.7 °C	
Butterflies	Nymphalidae	*Melitaea parthenoides*	0.80	10.5 °C	
Butterflies	Nymphalidae	*Melitaea phoebe*	0.60	10.9 °C	
Butterflies	Nymphalidae	*Melitaea varia*	0.80	5.6 °C	
Butterflies	Nymphalidae	*Minois dryas*	0.70	9.6 °C	
Butterflies	Nymphalidae	*Neptis rivularis*	0.90	7.7 °C	

Butterflies	Nymphalidae	*Nymphalis antiopa*	0.90	7.1 °C	
Butterflies	Nymphalidae	*Nymphalis polychloros*	0.90	9.9 °C	
Butterflies	Nymphalidae	*Oeneis glacialis*	0.70	4.8 °C	
Butterflies	Nymphalidae	*Pararge aegeria*	0.80	9.7 °C	
Butterflies	Nymphalidae	*Polygonia c-album*	0.90	8.4 °C	
Butterflies	Nymphalidae	*Pyronia tithonus*	0.70	10.8 °C	
Butterflies	Nymphalidae	*Satyrus ferula*	0.80	10.1 °C	
Butterflies	Nymphalidae	*Speyeria aglaja*	0.70	7.2 °C	
Butterflies	Nymphalidae	*Vanessa atalanta*	0.80	8.8 °C	migratory
Butterflies	Nymphalidae	*Vanessa cardui*	0.20	8.8 °C	migratory
Butterflies	Papilionidae	*Iphiclides podalirius*	0.60	11.0 °C	
Butterflies	Papilionidae	*Papilio machaon*	0.20	9.2 °C	
Butterflies	Papilionidae	*Parnassius apollo*	0.40	7.8 °C	
Butterflies	Papilionidae	*Parnassius mnemosyne*	0.40	7.8 °C	
Butterflies	Papilionidae	*Parnassius phoebus*	1.00	4.4 °C	
Butterflies	Pieridae	*Anthocharis cardamines*	0.50	8.2 °C	
Butterflies	Pieridae	*Aporia crataegi*	0.70	8.8 °C	
Butterflies	Pieridae	*Colias alfacariensis*	0.70	10.5 °C	difficult ID
Butterflies	Pieridae	*Colias crocea*	0.30	11.2 °C	migratory
Butterflies	Pieridae	*Colias hyale*	0.60	8.7 °C	difficult ID
Butterflies	Pieridae	*Colias palaeno*	0.80	3.5 °C	
Butterflies	Pieridae	*Colias phicomone*	0.70	6.5 °C	
Butterflies	Pieridae	*Euchloe simplonia*	0.60	8.3 °C	
Butterflies	Pieridae	*Gonepteryx rhamni*	0.90	8.5 °C	
Butterflies	Pieridae	*Leptidea juvernica*	0.50	6.6 °C	difficult ID
Butterflies	Pieridae	*Leptidea sinapis*	0.50	8.2 °C	difficult ID
Butterflies	Pieridae	*Pieris brassicae*	0.80	9.3 °C	
Butterflies	Pieridae	*Pieris bryoniae*	0.60	6.2 °C	
Butterflies	Pieridae	*Pieris mannii*	0.60	10.1 °C	
Butterflies	Pieridae	*Pieris napi*	0.40	7.8 °C	
Butterflies	Pieridae	*Pieris rapae*	0.60	9.5 °C	
Butterflies	Pieridae	*Pontia callidice*	0.80	5.7 °C	
Butterflies	Pieridae	*Pontia edusa*	0.80	9.6 °C	
Butterflies	Riodinidae	*Hamearis lucina*	0.60	9.2 °C	
Butterflies	Zygaenidae	*Adscita alpina*	0.30	4.6 °C	difficult ID
Butterflies	Zygaenidae	*Adscita dujardini*	0.60	6.1 °C	difficult ID
Butterflies	Zygaenidae	*Adscita geryon*	0.40	8.3 °C	difficult ID
Butterflies	Zygaenidae	*Adscita statices*	0.40	7.6 °C	difficult ID
Butterflies	Zygaenidae	*Jordanita globulariae*	0.50	9.8 °C	difficult ID
Butterflies	Zygaenidae	*Jordanita notata*	0.80	9.9 °C	difficult ID
Butterflies	Zygaenidae	*Jordanita subsolana*	0.60	8.7 °C	difficult ID
Butterflies	Zygaenidae	*Rhagades pruni*	0.80	8.0 °C	
Butterflies	Zygaenidae	*Zygaena carniolica*	0.60	9.5 °C	
Butterflies	Zygaenidae	*Zygaena ephialtes*	0.70	9.4 °C	
Butterflies	Zygaenidae	*Zygaena exulans*	0.80	2.2 °C	
Butterflies	Zygaenidae	*Zygaena fausta*	0.60	10.8 °C	
Butterflies	Zygaenidae	*Zygaena filipendulae*	0.20	8.7 °C	
Butterflies	Zygaenidae	*Zygaena lonicerae*	0.50	8.0 °C	
Butterflies	Zygaenidae	*Zygaena loti*	0.20	9.3 °C	
Butterflies	Zygaenidae	*Zygaena minos*	0.70	7.6 °C	
Butterflies	Zygaenidae	*Zygaena osterodensis*	0.80	7.0 °C	
Butterflies	Zygaenidae	*Zygaena purpuralis*	0.50	8.5 °C	
Butterflies	Zygaenidae	*Zygaena romeo*	0.70	8.9 °C	
Butterflies	Zygaenidae	*Zygaena transalpina*	0.30	9.6 °C	
Butterflies	Zygaenidae	*Zygaena trifolii*	1.00	10.1 °C	

Butterflies	Zygaenidae	*Zygaena viciae*	0.30	7.3 °C	
Grasshoppers	Acrididae	*Acrotylus patruelis*	0.91	15.1 °C	
Grasshoppers	Acrididae	*Aeropedellus variegatus*	0.95	3.0 °C	
Grasshoppers	Acrididae	*Aiolopus strepens*	0.45	12.8 °C	
Grasshoppers	Acrididae	*Aiolopus thalassinus*	0.59	11.9 °C	
Grasshoppers	Acrididae	*Anacridium aegyptium*	0.82	13.3 °C	
Grasshoppers	Acrididae	*Arcyptera fusca*	0.77	8.2 °C	
Grasshoppers	Acrididae	*Bohemanella frigida*	0.91	1.7 °C	
Grasshoppers	Acrididae	*Calliptamus barbarus*	0.86	12.7 °C	
Grasshoppers	Acrididae	*Calliptamus italicus*	0.59	10.8 °C	
Grasshoppers	Acrididae	*Calliptamus siciliae*	0.82	10.7 °C	
Grasshoppers	Acrididae	*Chorthippus albomarginatus*	0.68	8.9 °C	
Grasshoppers	Acrididae	*Chorthippus apricarius*	0.68	7.9 °C	
Grasshoppers	Acrididae	*Chorthippus biguttulus*	0.05	8.7 °C	
Grasshoppers	Acrididae	*Chorthippus brunneus*	0.00	8.4 °C	
Grasshoppers	Acrididae	*Chorthippus dorsatus*	0.64	8.9 °C	
Grasshoppers	Acrididae	*Chorthippus eisentrauti*	0.50	4.1 °C	
Grasshoppers	Acrididae	*Chorthippus mollis*	0.41	9.3 °C	
Grasshoppers	Acrididae	*Chorthippus montanus*	1.00	7.5 °C	
Grasshoppers	Acrididae	*Chorthippus parallelus*	0.14	8.8 °C	
Grasshoppers	Acrididae	*Chorthippus pullus*	1.00	5.2 °C	
Grasshoppers	Acrididae	*Chorthippus vagans*	0.59	10.7 °C	
Grasshoppers	Acrididae	*Chrysochraon dispar*	0.77	8.9 °C	
Grasshoppers	Acrididae	*Epacromius tergestinus*	1.00	9.5 °C	(re)introduced
Grasshoppers	Acrididae	*Euchorthippus declivus*	0.59	10.8 °C	
Grasshoppers	Acrididae	*Euthystira brachyptera*	0.55	8.0 °C	
Grasshoppers	Acrididae	*Gomphocerippus rufus*	0.18	8.0 °C	
Grasshoppers	Acrididae	*Gomphocerus sibiricus*	0.86	6.4 °C	
Grasshoppers	Acrididae	*Locusta migratoria*	0.82	12.0 °C	
Grasshoppers	Acrididae	*Mecostethus parapleurus*	0.68	9.3 °C	
Grasshoppers	Acrididae	*Miramella alpina*	0.50	6.9 °C	
Grasshoppers	Acrididae	*Miramella formosanta*	0.68	6.4 °C	
Grasshoppers	Acrididae	*Myrmeleotettix maculatus*	0.59	8.0 °C	
Grasshoppers	Acrididae	*Odontopodisma decipiens*	0.73	9.3 °C	
Grasshoppers	Acrididae	*Oedaleus decorus*	0.95	11.7 °C	
Grasshoppers	Acrididae	*Oedipoda caerulescens*	0.55	10.9 °C	
Grasshoppers	Acrididae	*Oedipoda germanica*	0.73	10.4 °C	
Grasshoppers	Acrididae	*Omocestus haemorrhoidalis*	0.77	8.8 °C	
Grasshoppers	Acrididae	*Omocestus rufipes*	0.59	10.3 °C	
Grasshoppers	Acrididae	*Omocestus viridulus*	0.73	7.5 °C	
Grasshoppers	Acrididae	*Pezotettix giornae*	0.59	12.6 °C	
Grasshoppers	Acrididae	*Podisma pedestris*	0.73	5.4 °C	
Grasshoppers	Acrididae	*Podismopsis keisti*	0.86	5.1 °C	
Grasshoppers	Acrididae	*Psophus stridulus*	0.77	7.4 °C	
Grasshoppers	Acrididae	*Sphingonotus caerulans*	0.77	10.2 °C	
Grasshoppers	Acrididae	*Stauroderus scalaris*	0.77	8.2 °C	
Grasshoppers	Acrididae	*Stenobothrus lineatus*	0.77	9.1 °C	
Grasshoppers	Acrididae	*Stenobothrus nigromaculatus*	0.73	8.9 °C	
Grasshoppers	Acrididae	*Stenobothrus rubicundulus*	0.82	7.4 °C	
Grasshoppers	Acrididae	*Stenobothrus stigmaticus*	0.86	9.7 °C	
Grasshoppers	Acrididae	*Stethophyma grossum*	1.00	7.7 °C	
Grasshoppers	Gryllidae	*Acheta domesticus*	0.95	8.9 °C	(re)introduced
Grasshoppers	Gryllidae	*Eumodicogryllus bordigalensis*	0.68	12.1 °C	
Grasshoppers	Gryllidae	*Gryllomorpha dalmatina*	0.95	12.3 °C	(re)introduced
Grasshoppers	Gryllidae	*Gryllus campestris*	0.68	10.1 °C	

Grasshoppers	Gryllidae	*Nemobius sylvestris*	0.59	10.3 °C	
Grasshoppers	Gryllidae	*Oecanthus pellucens*	0.32	10.9 °C	
Grasshoppers	Gryllidae	*Pteronemobius heydenii*	0.86	10.9 °C	
Grasshoppers	Gryllidae	*Pteronemobius lineolatus*	1.00	11.5 °C	
Grasshoppers	Gryllotalpidae	*Gryllotalpa gryllotalpa*	0.91	9.9 °C	
Grasshoppers	Tetrigidae	*Tetrix bipunctata*	0.32	6.0 °C	
Grasshoppers	Tetrigidae	*Tetrix ceperoi*	0.95	10.6 °C	
Grasshoppers	Tetrigidae	*Tetrix subulata*	0.41	8.4 °C	
Grasshoppers	Tetrigidae	*Tetrix tenuicornis*	0.55	9.1 °C	
Grasshoppers	Tetrigidae	*Tetrix tuerki*	1.00	6.2 °C	
Grasshoppers	Tetrigidae	*Tetrix undulata*	0.73	9.0 °C	
Grasshoppers	Tetrigidae	*Uvarovitettix depressus*	0.77	11.0 °C	
Grasshoppers	Tettigoniidae	*Anonconotus alpinus*	0.91	7.0 °C	
Grasshoppers	Tettigoniidae	*Antaxius difformis*	0.95	4.9 °C	
Grasshoppers	Tettigoniidae	*Antaxius pedestris*	0.45	8.3 °C	
Grasshoppers	Tettigoniidae	*Barbitistes obtusus*	0.73	8.1 °C	
Grasshoppers	Tettigoniidae	*Barbitistes serricauda*	0.77	8.4 °C	
Grasshoppers	Tettigoniidae	*Conocephalus dorsalis*	1.00	9.2 °C	
Grasshoppers	Tettigoniidae	*Conocephalus fuscus*	0.64	10.0 °C	
Grasshoppers	Tettigoniidae	*Decticus verrucivorus*	0.86	7.9 °C	
Grasshoppers	Tettigoniidae	*Ephippiger diurnus*	0.77	10.1 °C	
Grasshoppers	Tettigoniidae	*Ephippiger terrestris*	0.82	9.6 °C	
Grasshoppers	Tettigoniidae	*Ephippiger vicheti*	0.82	6.9 °C	
Grasshoppers	Tettigoniidae	*Eupholidoptera chabrieri*	0.59	11.4 °C	
Grasshoppers	Tettigoniidae	*Leptophyes albovittata*	0.82	8.3 °C	
Grasshoppers	Tettigoniidae	*Leptophyes laticauda*	0.73	10.6 °C	
Grasshoppers	Tettigoniidae	*Leptophyes punctatissima*	0.68	9.6 °C	
Grasshoppers	Tettigoniidae	*Meconema meridionale*	0.91	9.7 °C	
Grasshoppers	Tettigoniidae	*Meconema thalassinum*	0.95	9.2 °C	
Grasshoppers	Tettigoniidae	*Metrioptera bicolor*	0.91	8.9 °C	
Grasshoppers	Tettigoniidae	*Metrioptera brachyptera*	0.73	7.4 °C	
Grasshoppers	Tettigoniidae	*Metrioptera fedtschenkoi*	0.59	11.6 °C	
Grasshoppers	Tettigoniidae	*Metrioptera roeselii*	0.36	8.5 °C	
Grasshoppers	Tettigoniidae	*Metrioptera saussuriana*	0.82	8.3 °C	
Grasshoppers	Tettigoniidae	*Pachytrachis striolatus*	0.86	10.0 °C	
Grasshoppers	Tettigoniidae	*Phaneroptera falcata*	0.45	9.4 °C	
Grasshoppers	Tettigoniidae	*Phaneroptera nana*	0.45	11.7 °C	
Grasshoppers	Tettigoniidae	*Pholidoptera aptera*	0.64	7.3 °C	
Grasshoppers	Tettigoniidae	*Pholidoptera fallax*	0.68	9.9 °C	
Grasshoppers	Tettigoniidae	*Pholidoptera griseoaptera*	0.55	8.8 °C	
Grasshoppers	Tettigoniidae	*Pholidoptera littoralis*	0.91	8.9 °C	
Grasshoppers	Tettigoniidae	*Platycleis albopunctata*	0.55	9.7 °C	
Grasshoppers	Tettigoniidae	*Polysarcus denticauda*	0.73	8.5 °C	
Grasshoppers	Tettigoniidae	*Ruspolia nitidula*	0.32	11.1 °C	
Grasshoppers	Tettigoniidae	*Saga pedo*	0.91	11.2 °C	
Grasshoppers	Tettigoniidae	*Tettigonia cantans*	0.55	7.7 °C	
Grasshoppers	Tettigoniidae	*Tettigonia caudata*	0.86	9.1 °C	
Grasshoppers	Tettigoniidae	*Tettigonia viridissima*	0.27	9.8 °C	
Grasshoppers	Tettigoniidae	*Yersinella raymondii*	0.64	11.9 °C	
Dragonflies	Aeshnidae	*Aeshna affinis*	0.57	10.5 °C	
Dragonflies	Aeshnidae	*Aeshna caerulea*	0.79	2.4 °C	
Dragonflies	Aeshnidae	*Aeshna cyanea*	0.50	8.8 °C	
Dragonflies	Aeshnidae	*Aeshna grandis*	0.64	6.3 °C	
Dragonflies	Aeshnidae	*Aeshna isoceles*	0.57	10.2 °C	
Dragonflies	Aeshnidae	*Aeshna juncea*	0.43	5.6 °C	

Dragonflies	Aeshnidae	*Aeshna mixta*	0.36	9.9 °C	
Dragonflies	Aeshnidae	*Aeshna subarctica*	0.86	4.6 °C	
Dragonflies	Aeshnidae	*Anax ephippiger*	0.86	11.7 °C	migratory
Dragonflies	Aeshnidae	*Anax imperator*	0.36	10.4 °C	
Dragonflies	Aeshnidae	*Anax parthenope*	0.57	10.8 °C	
Dragonflies	Aeshnidae	*Boyeria irene*	0.93	11.9 °C	
Dragonflies	Aeshnidae	*Brachytron pratense*	0.57	8.9 °C	
Dragonflies	Calopterygidae	*Calopteryx splendens*	0.79	9.1 °C	
Dragonflies	Calopterygidae	*Calopteryx virgo*	0.79	8.6 °C	
Dragonflies	Coenagrionidae	*Ceriagrion tenellum*	0.57	11.6 °C	
Dragonflies	Coenagrionidae	*Coenagrion hastulatum*	0.71	4.7 °C	
Dragonflies	Coenagrionidae	*Coenagrion mercuriale*	0.79	11.3 °C	
Dragonflies	Coenagrionidae	*Coenagrion puella*	0.43	9.1 °C	
Dragonflies	Coenagrionidae	*Coenagrion pulchellum*	0.50	8.2 °C	
Dragonflies	Coenagrionidae	*Coenagrion scitulum*	0.43	11.4 °C	
Dragonflies	Coenagrionidae	*Enallagma cyathigerum*	0.43	8.0 °C	
Dragonflies	Coenagrionidae	*Erythromma lindenii*	0.64	11.5 °C	
Dragonflies	Coenagrionidae	*Erythromma najas*	0.64	7.6 °C	
Dragonflies	Coenagrionidae	*Erythromma viridulum*	0.64	10.3 °C	
Dragonflies	Coenagrionidae	*Ischnura elegans*	0.00	9.4 °C	
Dragonflies	Coenagrionidae	*Ischnura pumilio*	0.29	9.9 °C	
Dragonflies	Coenagrionidae	*Nehalennia speciosa*	0.86	6.9 °C	
Dragonflies	Coenagrionidae	*Pyrrhosoma nymphula*	0.43	8.7 °C	
Dragonflies	Cordulegastridae	*Cordulegaster bidentata*	0.79	8.8 °C	
Dragonflies	Cordulegastridae	*Cordulegaster boltonii*	0.86	8.7 °C	
Dragonflies	Corduliidae	*Cordulia aenea*	0.43	7.2 °C	
Dragonflies	Corduliidae	*Epitheca bimaculata*	0.86	7.4 °C	
Dragonflies	Corduliidae	*Oxygastra curtisii*	1.00	12.0 °C	
Dragonflies	Corduliidae	*Somatochlora alpestris*	0.64	2.4 °C	
Dragonflies	Corduliidae	*Somatochlora arctica*	0.64	4.5 °C	
Dragonflies	Corduliidae	*Somatochlora flavomaculata*	0.71	7.6 °C	
Dragonflies	Corduliidae	*Somatochlora metallica*	0.36	6.2 °C	
Dragonflies	Gomphidae	*Gomphus pulchellus*	0.71	10.7 °C	
Dragonflies	Gomphidae	*Gomphus simillimus*	1.00	12.2 °C	
Dragonflies	Gomphidae	*Gomphus vulgatissimus*	0.79	8.6 °C	
Dragonflies	Gomphidae	*Onychogomphus forcipatus*	0.79	9.7 °C	
Dragonflies	Gomphidae	*Ophiogomphus cecilia*	1.00	7.0 °C	
Dragonflies	Lestidae	*Chalcolestes viridis*	0.57	10.5 °C	
Dragonflies	Lestidae	*Lestes barbarus*	0.71	11.1 °C	
Dragonflies	Lestidae	*Lestes dryas*	0.50	8.8 °C	
Dragonflies	Lestidae	*Lestes sponsa*	0.57	7.5 °C	
Dragonflies	Lestidae	*Lestes virens*	0.71	10.1 °C	
Dragonflies	Lestidae	*Sympecma fusca*	0.64	10.4 °C	
Dragonflies	Lestidae	*Sympecma paedisca*	0.43	6.6 °C	
Dragonflies	Libellulidae	*Crocothemis erythraea*	0.79	11.7 °C	
Dragonflies	Libellulidae	*Leucorrhinia albifrons*	0.71	6.3 °C	
Dragonflies	Libellulidae	*Leucorrhinia caudalis*	0.71	7.0 °C	
Dragonflies	Libellulidae	*Leucorrhinia dubia*	0.79	4.8 °C	
Dragonflies	Libellulidae	*Leucorrhinia pectoralis*	0.86	7.6 °C	
Dragonflies	Libellulidae	*Libellula depressa*	0.71	9.4 °C	
Dragonflies	Libellulidae	*Libellula fulva*	0.71	9.9 °C	
Dragonflies	Libellulidae	*Libellula quadrimaculata*	0.36	7.7 °C	
Dragonflies	Libellulidae	*Orthetrum albistylum*	0.43	10.6 °C	
Dragonflies	Libellulidae	*Orthetrum brunneum*	0.50	11.3 °C	
Dragonflies	Libellulidae	*Orthetrum cancellatum*	0.50	10.0 °C	

Dragonflies	Libellulidae	*Orthetrum coerulescens*	0.79	10.5 °C	
Dragonflies	Libellulidae	*Sympetrum danae*	0.36	6.6 °C	
Dragonflies	Libellulidae	*Sympetrum depressiusculum*	0.64	9.4 °C	
Dragonflies	Libellulidae	*Sympetrum flaveolum*	0.64	7.4 °C	
Dragonflies	Libellulidae	*Sympetrum fonscolombii*	0.57	11.4 °C	migratory
Dragonflies	Libellulidae	*Sympetrum meridionale*	0.71	11.5 °C	
Dragonflies	Libellulidae	*Sympetrum pedemontanum*	0.43	8.8 °C	
Dragonflies	Libellulidae	*Sympetrum sanguineum*	0.36	9.0 °C	
Dragonflies	Libellulidae	*Sympetrum striolatum*	0.57	10.0 °C	
Dragonflies	Libellulidae	*Sympetrum vulgatum*	0.64	7.6 °C	
Dragonflies	Platycnemididae	*Platycnemis pennipes*	0.43	9.1 °C	

Table S2 Posterior distribution of fixed effect estimates from model linking climate and land-use changes as well as species traits to 5-year mean occupancy trends (Fig. 3a). First model version in which parameters for change in agricultural area and grassland-use intensity were only included for species of agriculturally influenced habitats (n = 13,968). 95% and 80% credible intervals (CIs) and means are shown. Colours code effect size on the same scale as in Fig. 3. n_{tot} = 20,048.

Variable	Lower 95%-CI	Lower 80%-CI	Mean	Upper 80%-CI	Upper 95%-CI
ΔT. Mean (low)	-0.0155	-0.0134	-0.0089	-0.0047	-0.0022
ΔT. Mean (high)	-0.0123	-0.0094	-0.0048	0.0001	0.0023
ΔT. Seasonality (low)	-0.0153	-0.0122	-0.0054	0.0009	0.0048
ΔT. Seasonality (high)	-0.0105	-0.0080	-0.0009	0.0052	0.0092
ΔP. Summer (low)	-0.0077	-0.0066	-0.0047	-0.0027	-0.0018
ΔP. Summer (high)	-0.0047	-0.0040	-0.0019	-0.0002	0.0010
ΔAgr. Area (low)	-0.0026	-0.0015	0.0000	0.0017	0.0024
ΔAgr. Area (high)	-0.0071	-0.0061	-0.0038	-0.0016	-0.0003
ΔGrassland int. (low)	-0.0033	-0.0022	-0.0006	0.0011	0.0017
ΔGrassland int. (high)	-0.0133	-0.0112	-0.0070	-0.0027	-0.0003
ΔCrop int.	-0.0011	-0.0002	0.0013	0.0027	0.0035
ΔT. Mean × ΔAgr. Area	-0.0065	-0.0055	-0.0039	-0.0021	-0.0014
ΔT. Mean × ΔGrassland int.	0.0027	0.0036	0.0056	0.0075	0.0087
ΔT. Mean × ΔCrop int.	-0.0019	-0.0012	-0.0002	0.0009	0.0014
ΔT. Seasonality × ΔAgr. Area	-0.0012	-0.0002	0.0017	0.0036	0.0045
ΔT. Seasonality × ΔGrassland int.	0.0051	0.0063	0.0089	0.0115	0.0131
ΔT. Seasonality × ΔCrop int.	-0.0048	-0.0042	-0.0031	-0.0019	-0.0013
ΔP. Summer × ΔAgr. Area	-0.0006	0.0001	0.0014	0.0029	0.0036
ΔP. Summer × ΔGrassland int.	0.0067	0.0078	0.0098	0.0118	0.0127
ΔP. Summer × ΔCrop int.	-0.0044	-0.0038	-0.0027	-0.0016	-0.0010
Temp. niche (low)	0.0047	0.0052	0.0069	0.0084	0.0093
Temp. niche (high)	0.0049	0.0055	0.0070	0.0084	0.0093
ΔT. Mean × Temp. niche	-0.0003	0.0005	0.0020	0.0035	0.0043
ΔT. Seasonality × Temp. niche	-0.0032	-0.0024	-0.0009	0.0005	0.0014
ΔP. Summer × Temp. niche	-0.0033	-0.0027	-0.0017	-0.0007	0.0000
Specialisation (low)	-0.0045	-0.0038	-0.0026	-0.0013	-0.0005
Specialisation (high)	-0.0056	-0.0049	-0.0034	-0.0022	-0.0014
ΔAgr. Area × Specialisation	-0.0039	-0.0033	-0.0023	-0.0012	-0.0007
ΔGrassland int. × Specialisation	0.0005	0.0009	0.0020	0.0030	0.0036
ΔCrop int. × Specialisation	-0.0018	-0.0014	-0.0007	0.0000	0.0004
Elevation (high)	-0.0045	-0.0021	0.0021	0.0063	0.0093
Intercept (butterflies)	-0.0381	-0.0336	-0.0238	-0.0145	-0.0089
Intercept (grasshoppers)	-0.0343	-0.0273	-0.0183	-0.0080	-0.0046
Intercept (dragonflies)	-0.0335	-0.0277	-0.0181	-0.0084	-0.0033
Interval 1985-1990	0.0115	0.0173	0.0286	0.0403	0.0473
Interval 1990-1995	0.0042	0.0115	0.0263	0.0414	0.0498
Interval 1995-2000	-0.0003	0.0068	0.0187	0.0317	0.0375
Interval 2000-2005	0.0408	0.0432	0.0477	0.0523	0.0550
Interval 2005-2010	-0.0095	-0.0053	0.0057	0.0161	0.0231
Interval 2010-2015	-0.0228	-0.0166	-0.0024	0.0115	0.0199
Interval 2015-2020	0.0273	0.0336	0.0425	0.0530	0.0578

ΔT. Mean: Annual mean temperature change; ΔT. Seasonality: Temperature seasonality change; ΔP. Summer: Summer precipitation change; ΔAgr. area: Agricultural area change; ΔGrassland int.: Grassland-use intensity change; ΔCrop int.: Crop-use intensity change; Temp. niche: Temperature niche

Table S3 Posterior distribution of fixed effect estimates from model linking climate and land-use changes as well as species traits to 5-year mean occupancy trends (Fig. 3a). Second model version in which only species of agriculturally influenced habitats were included. 95% and 80% credible intervals (CIs) and means are shown. Colours code effect size on the same scale as in Fig. 3. n_{tot} = 13,968.

Variable	Lower 95%-CI	Lower 80%-CI	Mean	Upper 80%-CI	Upper 95%-CI
ΔT. Mean (low)	-0.0203	-0.0169	-0.0116	-0.0055	-0.0034
ΔT. Mean (high)	-0.0195	-0.0161	-0.0102	-0.0036	-0.0005
ΔT. Seasonality (low)	-0.0272	-0.0217	-0.0136	-0.0045	-0.0005
ΔT. Seasonality (high)	-0.0234	-0.0171	-0.0089	0.0003	0.0037
ΔP. Summer (low)	-0.0055	-0.0041	-0.0016	0.0011	0.0024
ΔP. Summer (high)	-0.0039	-0.0025	-0.0002	0.0020	0.0032
ΔAgr. Area (low)	-0.0070	-0.0063	-0.0045	-0.0029	-0.0019
ΔAgr. Area (high)	-0.0114	-0.0103	-0.0076	-0.0052	-0.0035
ΔGrassland int. (low)	0.0020	0.0032	0.0049	0.0069	0.0077
ΔGrassland int. (high)	-0.0095	-0.0064	-0.0018	0.0034	0.0055
ΔCrop int.	-0.0016	-0.0006	0.0008	0.0024	0.0032
ΔT. Mean × ΔAgr. Area	-0.0075	-0.0063	-0.0045	-0.0026	-0.0017
ΔT. Mean × ΔGrassland int.	-0.0014	-0.0002	0.0022	0.0043	0.0058
ΔT. Mean × ΔCrop int.	-0.0004	0.0002	0.0016	0.0030	0.0039
ΔT. Seasonality × ΔAgr. Area	0.0009	0.0019	0.0041	0.0061	0.0071
ΔT. Seasonality × ΔGrassland int.	0.0045	0.0060	0.0089	0.0121	0.0138
ΔT. Seasonality × ΔCrop int.	-0.0071	-0.0063	-0.0047	-0.0032	-0.0024
ΔP. Summer × ΔAgr. Area	-0.0008	0.0000	0.0016	0.0032	0.0041
ΔP. Summer × ΔGrassland int.	0.0096	0.0106	0.0131	0.0154	0.0168
ΔP. Summer × ΔCrop int.	-0.0065	-0.0058	-0.0043	-0.0029	-0.0021
Temp. niche (low)	0.0030	0.0038	0.0059	0.0076	0.0088
Temp. niche (high)	0.0050	0.0059	0.0075	0.0093	0.0101
ΔT. Mean × Temp. niche	-0.0009	0.0001	0.0020	0.0038	0.0050
ΔT. Seasonality × Temp. niche	-0.0040	-0.0029	-0.0012	0.0009	0.0017
ΔP. Summer × Temp. niche	-0.0036	-0.0029	-0.0016	-0.0004	0.0003
Specialisation (low)	-0.0056	-0.0048	-0.0032	-0.0017	-0.0008
Specialisation (high)	-0.0065	-0.0056	-0.0040	-0.0025	-0.0016
ΔAgr. Area × Specialisation	-0.0037	-0.0032	-0.0022	-0.0012	-0.0006
ΔGrassland int. × Specialisation	0.0005	0.0010	0.0020	0.0030	0.0036
ΔCrop int. × Specialisation	-0.0017	-0.0012	-0.0003	0.0006	0.0012
Elevation (high)	-0.0057	-0.0037	0.0003	0.0037	0.0059
Intercept (butterflies)	-0.0205	-0.0161	-0.0041	0.0075	0.0160
Intercept (grasshoppers)	-0.0150	-0.0094	0.0029	0.0147	0.0223
Interval 1985-1990	-0.0038	0.0054	0.0200	0.0355	0.0419
Interval 1990-1995	-0.0229	-0.0105	0.0080	0.0280	0.0371
Interval 1995-2000	-0.0241	-0.0119	0.0045	0.0207	0.0267
Interval 2000-2005	0.0279	0.0311	0.0373	0.0433	0.0463
Interval 2005-2010	-0.0535	-0.0452	-0.0303	-0.0164	-0.0088
Interval 2010-2015	-0.0692	-0.0581	-0.0406	-0.0216	-0.0121
Interval 2015-2020	0.0078	0.0150	0.0271	0.0404	0.0464

ΔT. Mean: Annual mean temperature change; ΔT. Seasonality: Temperature seasonality change; ΔP. Summer: Summer precipitation change; ΔAgr. area: Agricultural area change; ΔGrassland int.: Grassland-use intensity change; ΔCrop int.: Crop-use intensity change; Temp. niche: Temperature niche

Table S4 Posterior distribution of fixed effect estimates from model linking climate and land-use changes as well as species traits to 5-year mean occupancy trends (Fig. 3a). Third model version in which all species were included in the estimation of all parameters. 95% and 80% credible intervals (CIs) and means are shown. Colours code effect size on the same scale as in Fig. 3. n_{tot} = 20,048.

Variable	Lower 95%-CI	Lower 80%-CI	Mean	Upper 80%-CI	Upper 95%-CI
ΔT. Mean (low)	-0.0144	-0.0121	-0.0073	-0.0028	-0.0004
ΔT. Mean (high)	-0.0158	-0.0131	-0.0083	-0.0030	0.0000
ΔT. Seasonality (low)	-0.0238	-0.0204	-0.0130	-0.0065	-0.0027
ΔT. Seasonality (high)	-0.0227	-0.0190	-0.0117	-0.0050	-0.0009
ΔP. Summer (low)	-0.0048	-0.0037	-0.0015	0.0006	0.0017
ΔP. Summer (high)	-0.0052	-0.0041	-0.0023	-0.0003	0.0005
ΔAgr. Area (low)	-0.0068	-0.0060	-0.0046	-0.0031	-0.0024
ΔAgr. Area (high)	-0.0089	-0.0076	-0.0055	-0.0033	-0.0023
ΔGrassland int. (low)	0.0025	0.0033	0.0050	0.0064	0.0073
ΔGrassland int. (high)	-0.0103	-0.0078	-0.0034	0.0004	0.0027
ΔCrop int.	-0.0024	-0.0016	-0.0002	0.0011	0.0017
ΔT. Mean × ΔAgr. Area	-0.0044	-0.0036	-0.0021	-0.0006	0.0003
ΔT. Mean × ΔGrassland int.	0.0001	0.0008	0.0028	0.0046	0.0057
ΔT. Mean × ΔCrop int.	-0.0005	0.0000	0.0011	0.0023	0.0029
ΔT. Seasonality × ΔAgr. Area	0.0036	0.0045	0.0062	0.0078	0.0088
ΔT. Seasonality × ΔGrassland int.	0.0068	0.0081	0.0106	0.0129	0.0142
ΔT. Seasonality × ΔCrop int.	-0.0064	-0.0057	-0.0045	-0.0033	-0.0027
ΔP. Summer × ΔAgr. Area	0.0002	0.0008	0.0021	0.0034	0.0041
ΔP. Summer × ΔGrassland int.	0.0084	0.0094	0.0113	0.0131	0.0142
ΔP. Summer × ΔCrop int.	-0.0060	-0.0054	-0.0042	-0.0031	-0.0025
Temp. niche (low)	0.0043	0.0051	0.0067	0.0082	0.0092
Temp. niche (high)	0.0049	0.0056	0.0071	0.0086	0.0094
ΔT. Mean × Temp. niche	-0.0002	0.0007	0.0021	0.0037	0.0043
ΔT. Seasonality × Temp. niche	-0.0031	-0.0023	-0.0008	0.0007	0.0015
ΔP. Summer × Temp. niche	-0.0031	-0.0025	-0.0015	-0.0004	0.0001
Specialisation (low)	-0.0044	-0.0037	-0.0024	-0.0011	-0.0005
Specialisation (high)	-0.0051	-0.0043	-0.0030	-0.0015	-0.0010
ΔAgr. Area × Specialisation	-0.0031	-0.0025	-0.0017	-0.0008	-0.0003
ΔGrassland int. × Specialisation	0.0001	0.0007	0.0015	0.0024	0.0027
ΔCrop int. × Specialisation	-0.0018	-0.0014	-0.0007	0.0000	0.0005
Elevation (high)	-0.0037	-0.0014	0.0024	0.0054	0.0081
Intercept (butterflies)	-0.0239	-0.0181	-0.0091	0.0013	0.0060
Intercept (grasshoppers)	-0.0193	-0.0139	-0.0039	0.0058	0.0113
Intercept (dragonflies)	-0.0175	-0.0116	-0.0015	0.0081	0.0133
Interval 1985-1990	-0.0054	0.0022	0.0140	0.0262	0.0317
Interval 1990-1995	-0.0165	-0.0081	0.0070	0.0231	0.0309
Interval 1995-2000	-0.0149	-0.0086	0.0041	0.0173	0.0254
Interval 2000-2005	0.0330	0.0354	0.0405	0.0454	0.0483
Interval 2005-2010	-0.0335	-0.0275	-0.0155	-0.0044	0.0028
Interval 2010-2015	-0.0516	-0.0436	-0.0286	-0.0142	-0.0058
Interval 2015-2020	0.0088	0.0143	0.0253	0.0350	0.0407

ΔT. Mean: Annual mean temperature change; ΔT. Seasonality: Temperature seasonality change; ΔP. Summer: Summer precipitation change; ΔAgr. area: Agricultural area change; ΔGrassland int.: Grassland-use intensity change; ΔCrop int.: Crop-use intensity change; Temp. niche: Temperature niche

Table S5 Overview of priors used for occupancy-detection models and the model for 5-year interval species trends.

Model	Parameter	Meaning	Prior
Occupancy-detection	μ_o	global intercept (occ.)	$\mathrm{Normal}(0, 1.5^2)$
Occupancy-detection	$\sigma_{\gamma r}$	SD of year random effect (t>1) (occ.)	$\mathrm{Cauchy}(0,1)$
Occupancy-detection	$\gamma_{r,1}$	year 1 random effect (occ.)	$\mathrm{Normal}(0, 1.5^2)$
Occupancy-detection	σ_o	SD of random effects (occ.)	$\mathrm{Cauchy}(0,1)$
Occupancy-detection	β_o	slope fixed effects (occ.)	$\mathrm{Normal}(0, 5^2)$
Occupancy-detection	μ_d	global intercept (det.)	$\mathrm{Normal}(0, 1.5^2)$
Occupancy-detection	σ_d	SD of random effects (det.)	$\mathrm{Cauchy}(0,1)$
Occupancy-detection	β_d	slope fixed effects (det.)	$\mathrm{Normal}(0, 5^2)$
Trends	σ_r	SD of random effects	$\mathrm{Cauchy}(0,1)$
Trends	α_r	random effects	$\mathrm{Normal}(0, {\sigma_r}^2)$
Trends	μ	global intercept	$\mathrm{Normal}(\bar{y}, 5^2)$
Trends	β	slope fixed effects	$\mathrm{Normal}(0, 5^2)$
Trends	σ_y	SD of residuals	$\mathrm{Cauchy}(0,25)$

occ.: occurrence probability model
det.: detection probability model

Table S6 Posterior distribution of fixed effect estimates from model linking climate and land-use changes as well as species traits to 10-year mean occupancy trends. Species trends as well as climate and land-use variables were determined for four consecutive 10-year intervals. In contrast to analyses of 5-year intervals, climate change variables did not include the five preceding years. First model version in which parameters for change in agricultural area and grassland-use intensity were only included for species of agriculturally influenced habitats (n = 6,984). 95% and 80% credible intervals (CIs) and means are shown. Colours code effect size on the same scale as in Fig. 3. n_{tot} = 10,024.

Variable	Lower 95%-CI	Lower 80%-CI	Mean	Upper 80%-CI	Upper 95%-CI
ΔT. Mean (low)	-0.0340	-0.0271	-0.0130	0.0017	0.0089
ΔT. Mean (high)	-0.0486	-0.0430	-0.0304	-0.0195	-0.0125
ΔT. Seasonality (low)	-0.0150	-0.0119	-0.0058	0.0005	0.0038
ΔT. Seasonality (high)	-0.0355	-0.0319	-0.0240	-0.0173	-0.0135
ΔP. Summer (low)	-0.0149	-0.0127	-0.0084	-0.0046	-0.0027
ΔP. Summer (high)	-0.0164	-0.0145	-0.0116	-0.0085	-0.0072
ΔAgr. Area (low)	-0.0044	-0.0030	-0.0004	0.0020	0.0034
ΔAgr. Area (high)	-0.0170	-0.0154	-0.0120	-0.0090	-0.0072
ΔGrassland int. (low)	-0.0195	-0.0176	-0.0141	-0.0106	-0.0086
ΔGrassland int. (high)	-0.0219	-0.0188	-0.0136	-0.0080	-0.0058
ΔCrop int.	-0.0009	0.0003	0.0028	0.0050	0.0066
ΔT. Mean × ΔAgr. Area	0.0021	0.0040	0.0074	0.0109	0.0126
ΔT. Mean × ΔGrassland int.	-0.0072	-0.0049	-0.0010	0.0033	0.0054
ΔT. Mean × ΔCrop int.	-0.0171	-0.0148	-0.0116	-0.0076	-0.0063
ΔT. Seasonality × ΔAgr. Area	-0.0007	0.0013	0.0050	0.0086	0.0104
ΔT. Seasonality × ΔGrassland int.	-0.0079	-0.0056	-0.0026	0.0013	0.0026
ΔT. Seasonality × ΔCrop int.	-0.0135	-0.0119	-0.0082	-0.0048	-0.0026
ΔP. Summer × ΔAgr. Area	0.0020	0.0033	0.0062	0.0089	0.0104
ΔP. Summer × ΔGrassland int.	0.0119	0.0139	0.0178	0.0219	0.0241
ΔP. Summer × ΔCrop int.	-0.0110	-0.0099	-0.0081	-0.0060	-0.0053
Temp. niche (low)	0.0050	0.0062	0.0078	0.0096	0.0103
Temp. niche (high)	0.0042	0.0051	0.0067	0.0084	0.0093
ΔT. Mean × Temp. niche	-0.0065	-0.0051	-0.0026	0.0000	0.0015
ΔT. Seasonality × Temp. niche	-0.0060	-0.0049	-0.0025	-0.0004	0.0009
ΔP. Summer × Temp. niche	-0.0080	-0.0072	-0.0057	-0.0041	-0.0034
Specialisation (low)	-0.0023	-0.0015	-0.0001	0.0014	0.0023
Specialisation (high)	-0.0066	-0.0057	-0.0041	-0.0025	-0.0018
ΔAgr. Area × Specialisation	-0.0027	-0.0021	-0.0009	0.0002	0.0008
ΔGrassland int. × Specialisation	0.0001	0.0008	0.0020	0.0031	0.0038
ΔCrop int. × Specialisation	-0.0012	-0.0007	0.0002	0.0010	0.0014
Elevation (high)	-0.0068	-0.0026	0.0030	0.0083	0.0116
Intercept (butterflies)	-0.0206	-0.0149	-0.0030	0.0079	0.0138
Intercept (grasshoppers)	-0.0161	-0.0107	0.0009	0.0125	0.0186
Intercept (dragonflies)	-0.0143	-0.0096	0.0020	0.0128	0.0203
Interval 1990-2000	-0.0278	-0.0202	-0.0050	0.0097	0.0166
Interval 2000-2010	-0.0201	-0.0091	0.0121	0.0356	0.0461
Interval 2010-2020	-0.0095	-0.0061	0.0007	0.0076	0.0112

ΔT. Mean: Annual mean temperature change; ΔT. Seasonality: Temperature seasonality change; ΔP. Summer: Summer precipitation change; ΔAgr. area: Agricultural area change; ΔGrassland int.: Grassland-use intensity change; ΔCrop int.: Crop-use intensity change; Temp. niche: Temperature niche

Table S7 Availability of data from agricultural statistics and censuses.

Variable	Available years
Total agricultural area	1955[3], 1965[3], 1975[4], 1980[4], 1985[4], 1990[4], 1996–2020[4]
Grassland area[1]	1955 (r.), 1965 (r.), 1975[4], 1980[4], 1985[4], 1990[4], 1996–2020[4]
Crops	1969[3], 1975[4], 1980[4], 1985[4], 1990[4], 1996–2020[4]
Livestock units[2]	1956[3] (r.), 1966[3] (r.), 1975[4], 1978[3] (r.), 1980[4], 1985[4], 1990[4], 1996–2020[4]

[1] Grassland area was reconstructed (r.) for early years from total agricultural area and the proportion of grassland area therein in 1975.

[2] For some years, only numbers of cattle were available. Number of total livestock units was reconstructed (r.) from the relation between total livestock and cattle numbers in other years while accounting for municipality identity.

[3] Data from historical reports:

Bundesamt für Statistik. (1959). Bodenbenützung in der Schweiz 1955 (Statistische Quellenwerke der Schweiz 307, 402 pages).

Bundesamt für Statistik. (1960). Nutztierbestand der Schweiz 1956 (Statistische Quellenwerke der Schweiz 312, 322 pages).

Bundesamt für Statistik. (1967). Bodenbenützung 1965 (Statistische Quellenwerke der Schweiz 416, 132 pages).

Bundesamt für Statistik. (1968). Nutztierbestand der Schweiz 1966 (Statistische Quellenwerke der Schweiz 421, 485 pages).

Bundesamt für Statistik. (1970). Bodenbenützung 1969 (Statistische Quellenwerke der Schweiz 450, 288 pages).

Bundesamt für Statistik. (1979). Nutztierbestand der Schweiz 1978 (Statistische Quellenwerke der Schweiz 635, 369 pages).

(Most reports are available from https://www.bfs.admin.ch/bfs/de/home/dienstleistungen/historische-daten/publikationen.html)

[4] Data available from Bundesamt für Statistik through https://www.pxweb.bfs.admin.ch/pxweb/en/px-x-0702000000_104/-/px-x-0702000000_104.px. Data on crops only available upon request.

Table S8 Typical habitats for the three studied insect groups as defined in ref.[1], based on which a habitat specialisation index for each species was determined. The same habitat classification was used to define species occurring in agriculturally influenced habitats (highlighted in bold).

Group	Category	Habitat
Butterflies	Grasslands	**Lowland mesic grasslands**
		**Lowland semi-dry grasslands**
		**Lowland dry grasslands**
		Lowland rocky outcrops
		**Subalpine mesic grasslands**
		**Subalpine dry grasslands**
		Subalpine rocky outcrops
		**Alpine tall grasslands**
		**Alpine short grasslands**
		Alpine rocky outcrops
	Wetlands	**Lowland tall forb communities**
		**Lowland fens**
		Lowland peat bogs
		**Subalpine tall forb communities**
		**Subalpine bogs and fens**
		Spring areas, shores of creeks
	Woodlands	Lowland shrubs
		Lowland forests
		Dwarf shrubs
		Subalpine forests
	Other	**Ruderal habitats, roadsides, field margins, etc.**
		**Crop fields**
		**Fallows**
		Screes
Grasshoppers	Grasslands	**Leys**
		**Nutrient-rich, intensive grasslands**
		**Extensive grasslands**
		**Short grasslands**
		**Nutrient-poor grasslands**
		**Semi-dry grasslands**
		**Dry grasslands**
		**Alpine grasslands**
		**Alpine pastures**
		Rocky outcrops
	Wetlands	**Tall forb communities**
		**Fens**
		Peat bogs
		Marshes along water bodies
		Shores
		Gravel and sand banks
	Woodlands	Forest edges
		Windthrow areas
		Forest clearings
		Herbaceous fringes
		Shrubs
		Hedges
		Dwarf shrubs
	Other	Caves
		Cellars, garages
		Building walls
		**Crop fields**
		**Fallows**
		**Ruderal habitats**
		Railway areas
		Abandoned quarries

		Gravel pits
		Roadsides
		**Vineyards**
		Screes
		Rocks
Dragonflies	Lentic waters	Lakes
		Okbow lakes
		Small pioneer waters
		Small lakes
		Ponds
		Artificial ponds
		Small pools
		Depressions, hollows
		Puddles, ruts
		Deer and wild boar wallows
		Gravel pits
		Clay pits
		Quarry ponds
	Lotic waters	Rivers
		Creeks
		Canals
		Ditches
		Limnocrene
		Rheocrene
		Helocrene
	Wetlands	Peat bogs
		Transition mires
		Fens
		Slope bogs
		Bog waters

Table S9 Posterior distribution of fixed effect estimates from model linking climate and land-use changes as well as species traits to 5-year mean occupancy trends. Sensitivity analysis when critical species (migratory, (re)introduced, uncertain taxonomic status, difficult identification) and species with few records (lower 20% of each group) are excluded from analyses. First model version in which parameters for change in agricultural area and grassland-use intensity were only included for species of agriculturally influenced habitats (n = 12,688). 95% and 80% credible intervals (CIs) and means are shown. Colours code effect size on the same scale as in Fig. 3. n_{tot} = 17,936.

Variable	Lower 95%-CI	Lower 80%-CI	Mean	Upper 80%-CI	Upper 95%-CI
ΔT. Mean (low)	-0.0178	-0.0152	-0.0101	-0.0053	-0.0026
ΔT. Mean (high)	-0.0140	-0.0117	-0.0060	-0.0007	0.0029
ΔT. Seasonality (low)	-0.0172	-0.0131	-0.0058	0.0014	0.0056
ΔT. Seasonality (high)	-0.0128	-0.0087	-0.0011	0.0061	0.0100
ΔP. Summer (low)	-0.0084	-0.0071	-0.0049	-0.0027	-0.0017
ΔP. Summer (high)	-0.0051	-0.0040	-0.0021	0.0002	0.0011
ΔAgr. Area (low)	-0.0029	-0.0023	-0.0004	0.0013	0.0025
ΔAgr. Area (high)	-0.0080	-0.0066	-0.0043	-0.0018	-0.0007
ΔGrassland int. (low)	-0.0033	-0.0024	-0.0006	0.0012	0.0021
ΔGrassland int. (high)	-0.0140	-0.0120	-0.0075	-0.0032	-0.0007
ΔCrop int.	-0.0017	-0.0007	0.0009	0.0024	0.0033
ΔT. Mean × ΔAgr. Area	-0.0071	-0.0061	-0.0042	-0.0024	-0.0015
ΔT. Mean × ΔGrassland int.	0.0022	0.0035	0.0055	0.0079	0.0090
ΔT. Mean × ΔCrop int.	-0.0022	-0.0017	-0.0004	0.0007	0.0015
ΔT. Seasonality × ΔAgr. Area	-0.0013	-0.0003	0.0017	0.0036	0.0048
ΔT. Seasonality × ΔGrassland int.	0.0045	0.0064	0.0091	0.0120	0.0132
ΔT. Seasonality × ΔCrop int.	-0.0050	-0.0044	-0.0031	-0.0019	-0.0011
ΔP. Summer × ΔAgr. Area	-0.0009	-0.0001	0.0014	0.0030	0.0038
ΔP. Summer × ΔGrassland int.	0.0070	0.0082	0.0103	0.0126	0.0137
ΔP. Summer × ΔCrop int.	-0.0049	-0.0043	-0.0032	-0.0020	-0.0012
Temp. niche (low)	0.0060	0.0072	0.0089	0.0107	0.0115
Temp. niche (high)	0.0065	0.0072	0.0090	0.0106	0.0116
ΔT. Mean × Temp. niche	-0.0010	0.0002	0.0018	0.0038	0.0045
ΔT. Seasonality × Temp. niche	-0.0035	-0.0025	-0.0007	0.0011	0.0020
ΔP. Summer × Temp. niche	-0.0041	-0.0032	-0.0021	-0.0008	-0.0003
Specialisation (low)	-0.0040	-0.0033	-0.0020	-0.0005	0.0002
Specialisation (high)	-0.0056	-0.0047	-0.0033	-0.0018	-0.0010
ΔAgr. Area × Specialisation	-0.0040	-0.0035	-0.0024	-0.0014	-0.0007
ΔGrassland int. × Specialisation	0.0006	0.0012	0.0022	0.0033	0.0038
ΔCrop int. × Specialisation	-0.0018	-0.0014	-0.0006	0.0002	0.0006
Elevation (high)	-0.0064	-0.0025	0.0015	0.0061	0.0087
Intercept (butterflies)	-0.0381	-0.0329	-0.0220	-0.0116	-0.0060
Intercept (grasshoppers)	-0.0328	-0.0272	-0.0168	-0.0057	0.0001
Intercept (dragonflies)	-0.0330	-0.0257	-0.0158	-0.0042	-0.0003
Interval 1985-1990	0.0125	0.0160	0.0303	0.0430	0.0528
Interval 1990-1995	0.0016	0.0100	0.0265	0.0441	0.0535
Interval 1995-2000	-0.0057	0.0030	0.0162	0.0312	0.0372
Interval 2000-2005	0.0408	0.0438	0.0490	0.0544	0.0573
Interval 2005-2010	-0.0159	-0.0087	0.0022	0.0152	0.0214
Interval 2010-2015	-0.0277	-0.0211	-0.0044	0.0108	0.0205
Interval 2015-2020	0.0262	0.0319	0.0431	0.0545	0.0601

ΔT. Mean: Annual mean temperature change; ΔT. Seasonality: Temperature seasonality change; ΔP. Summer: Summer precipitation change; ΔAgr. area: Agricultural area change; ΔGrassland int.: Grassland-use intensity change; ΔCrop int.: Crop-use intensity change; Temp. niche: Temperature niche

Table S10 Posterior distribution of fixed effect estimates from model linking climate and land-use changes as well as species traits to 5-year mean occupancy trends. Sensitivity analysis when species-region combinations with very few records (< 41) are excluded from analyses. First model version in which parameters for change in agricultural area and grassland-use intensity were only included for species of agriculturally influenced habitats (n = 10,256). 95% and 80% credible intervals (CIs) and means are shown. Colours code effect size on the same scale as in Fig. 3. n_{tot} = 14,136.

Variable	Lower 95%-CI	Lower 80%-CI	Mean	Upper 80%-CI	Upper 95%-CI
ΔT. Mean (low)	-0.0283	-0.0251	-0.0190	-0.0132	-0.0100
ΔT. Mean (high)	-0.0186	-0.0154	-0.0088	-0.0023	0.0016
ΔT. Seasonality (low)	-0.0291	-0.0246	-0.0160	-0.0072	-0.0019
ΔT. Seasonality (high)	-0.0205	-0.0154	-0.0068	0.0022	0.0069
ΔP. Summer (low)	-0.0127	-0.0113	-0.0085	-0.0059	-0.0043
ΔP. Summer (high)	-0.0074	-0.0058	-0.0033	-0.0006	0.0007
ΔAgr. Area (low)	-0.0042	-0.0030	-0.0011	0.0010	0.0018
ΔAgr. Area (high)	-0.0113	-0.0095	-0.0064	-0.0032	-0.0015
ΔGrassland int. (low)	-0.0018	-0.0006	0.0015	0.0036	0.0048
ΔGrassland int. (high)	-0.0193	-0.0171	-0.0113	-0.0063	-0.0030
ΔCrop int.	-0.0008	-0.0001	0.0015	0.0030	0.0041
ΔT. Mean × ΔAgr. Area	-0.0104	-0.0092	-0.0069	-0.0047	-0.0035
ΔT. Mean × ΔGrassland int.	0.0022	0.0036	0.0063	0.0090	0.0105
ΔT. Mean × ΔCrop int.	-0.0029	-0.0022	-0.0007	0.0006	0.0015
ΔT. Seasonality × ΔAgr. Area	-0.0030	-0.0017	0.0008	0.0033	0.0046
ΔT. Seasonality × ΔGrassland int.	0.0056	0.0075	0.0110	0.0144	0.0164
ΔT. Seasonality × ΔCrop int.	-0.0074	-0.0064	-0.0050	-0.0032	-0.0025
ΔP. Summer × ΔAgr. Area	-0.0029	-0.0018	0.0002	0.0020	0.0030
ΔP. Summer × ΔGrassland int.	0.0086	0.0100	0.0125	0.0152	0.0164
ΔP. Summer × ΔCrop int.	-0.0058	-0.0050	-0.0036	-0.0021	-0.0015
Temp. niche (low)	0.0077	0.0091	0.0113	0.0137	0.0148
Temp. niche (high)	0.0087	0.0100	0.0118	0.0140	0.0148
ΔT. Mean × Temp. niche	0.0015	0.0025	0.0046	0.0067	0.0079
ΔT. Seasonality × Temp. niche	-0.0034	-0.0024	-0.0002	0.0017	0.0028
ΔP. Summer × Temp. niche	-0.0033	-0.0025	-0.0008	0.0008	0.0017
Specialisation (low)	-0.0049	-0.0042	-0.0025	-0.0011	-0.0001
Specialisation (high)	-0.0053	-0.0043	-0.0026	-0.0009	0.0001
ΔAgr. Area × Specialisation	-0.0042	-0.0036	-0.0024	-0.0010	-0.0003
ΔGrassland int. × Specialisation	0.0002	0.0008	0.0021	0.0033	0.0040
ΔCrop int. × Specialisation	-0.0021	-0.0015	-0.0007	0.0004	0.0007
Elevation (high)	0.0007	0.0035	0.0073	0.0111	0.0133
Intercept (butterflies)	-0.0363	-0.0306	-0.0176	-0.0054	0.0023
Intercept (grasshoppers)	-0.0300	-0.0222	-0.0095	0.0033	0.0096
Intercept (dragonflies)	-0.0262	-0.0195	-0.0061	0.0059	0.0130
Interval 1985-1990	0.0080	0.0189	0.0347	0.0508	0.0574
Interval 1990-1995	-0.0098	-0.0006	0.0210	0.0403	0.0525
Interval 1995-2000	-0.0173	-0.0080	0.0091	0.0255	0.0335
Interval 2000-2005	0.0472	0.0508	0.0567	0.0635	0.0668
Interval 2005-2010	-0.0334	-0.0265	-0.0119	0.0024	0.0106
Interval 2010-2015	-0.0563	-0.0446	-0.0273	-0.0066	0.0018
Interval 2015-2020	0.0190	0.0274	0.0400	0.0543	0.0600

ΔT. Mean: Annual mean temperature change; ΔT. Seasonality: Temperature seasonality change; ΔP. Summer: Summer precipitation change; ΔAgr. area: Agricultural area change; ΔGrassland int.: Grassland-use intensity change; ΔCrop int.: Crop-use intensity change; Temp. niche: Temperature niche

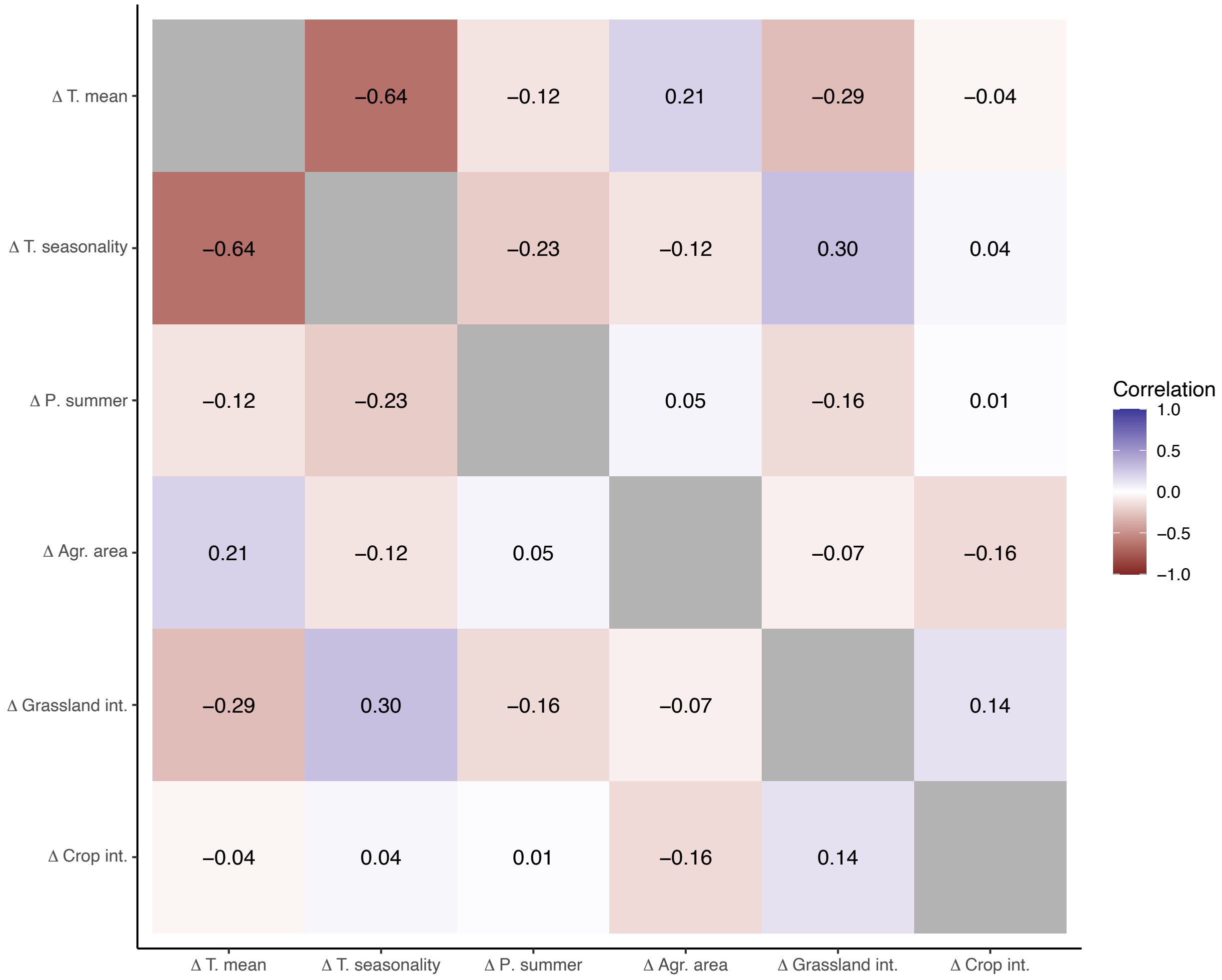

Fig. S1 Correlations of climate and land-use change variables. Pearson correlations of the three climate change variables (annual mean temperature, temperature seasonality, summer precipitation; linear trend estimates in the respective 5-year interval and the preceding 5 years) and three land-use variables (total agricultural area, grassland-use intensity, crop-use intensity; change across the 5 focal years). Separate values were determined per bioclimatic zone (n = 9) and time interval (n = 8). n_{tot} = 72.

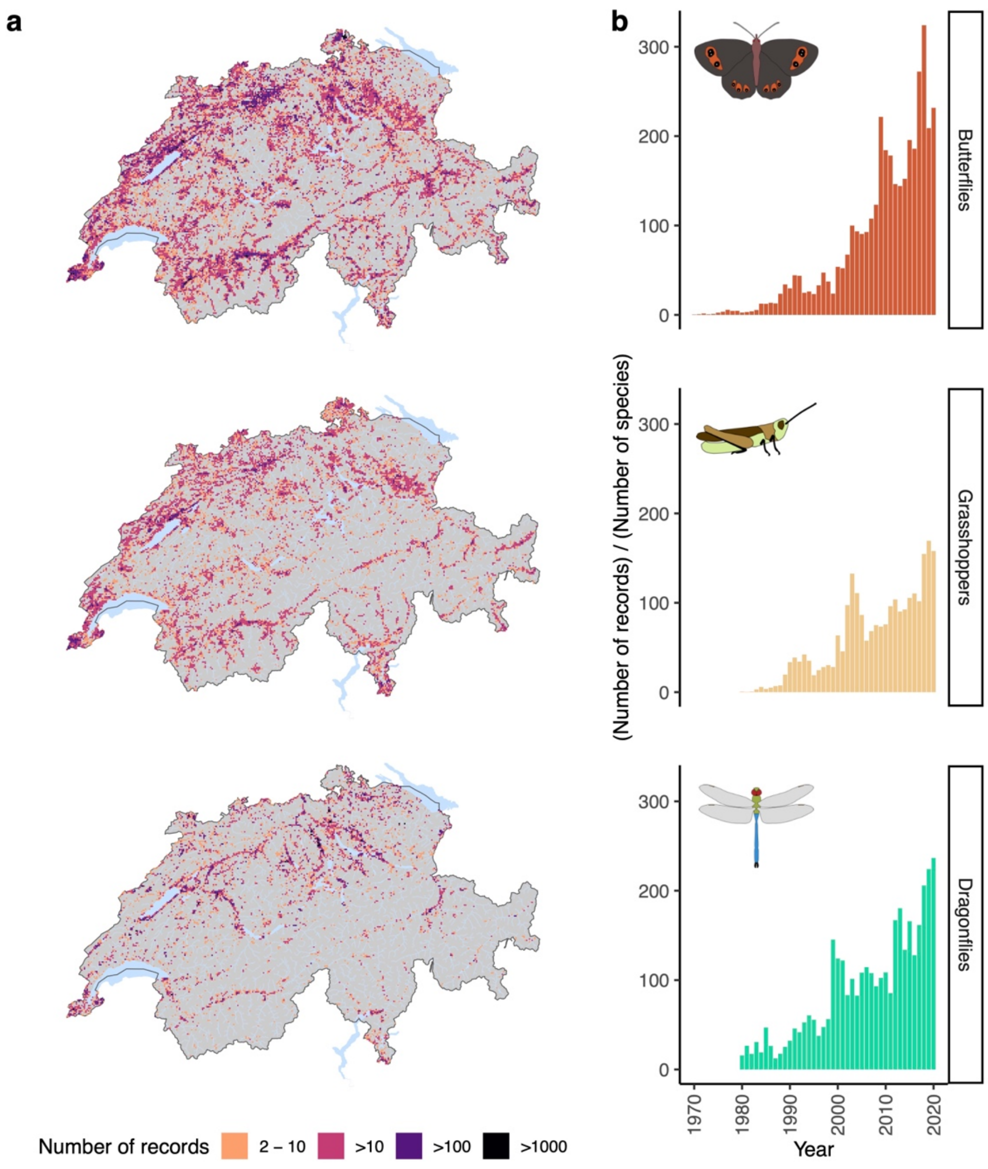

Fig. S2 Spatial and temporal distribution of records. a, Map of Switzerland with all squares (1 km ×1 km) that were analysed per insect group (butterflies, grasshoppers, dragonflies). The colour of the squares denotes the number of records (i.e. unique combinations of species and visit) per square. Waterbodies are shown in light blue. **b**, Number of records per year, standardised by dividing through the total number of species analysed per group (215 for butterflies, 103 for grasshoppers, 72 for dragonflies). Note that only for occupancy-detection models of butterflies, data from 1970–1979 were included. Mean occupancy estimates for these years were later excluded from subsequent analyses.

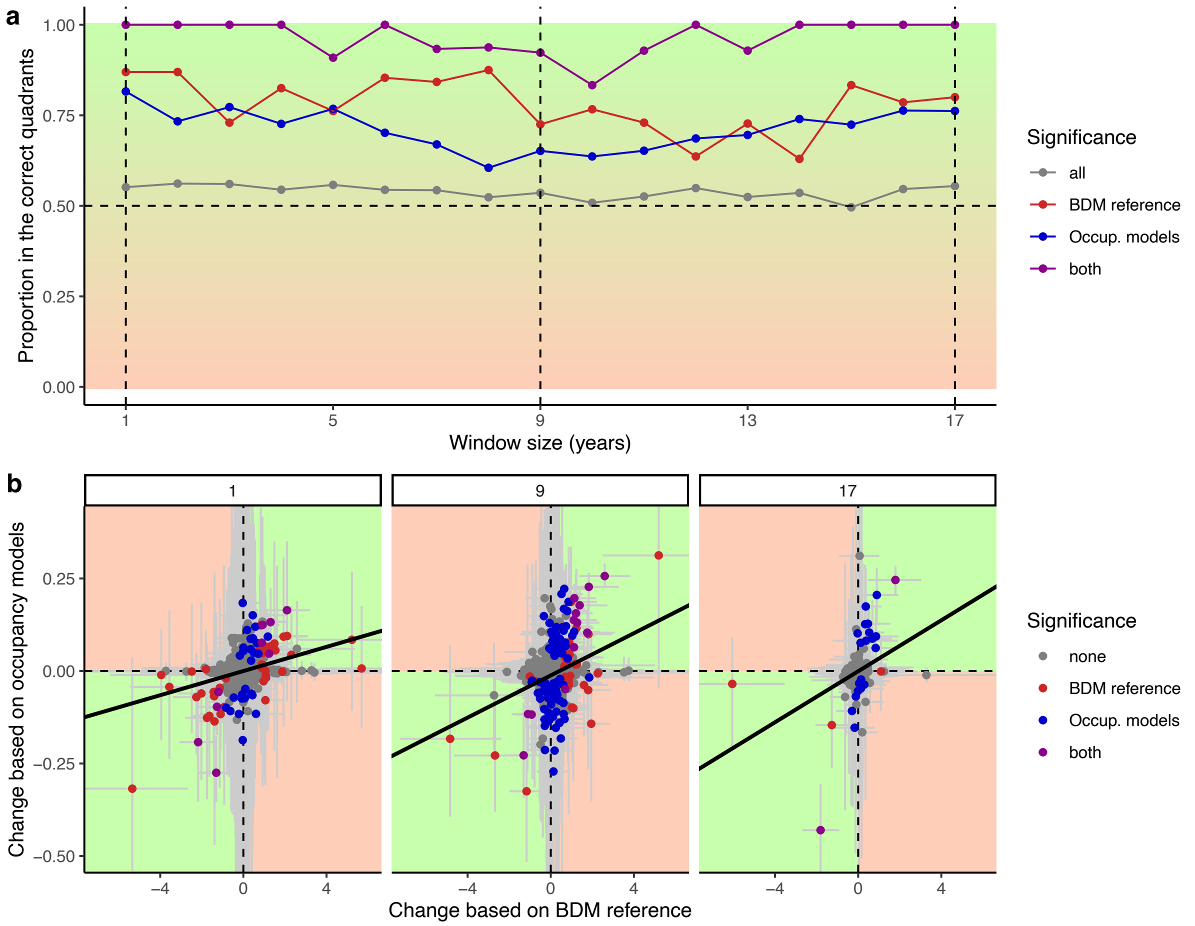

Fig. S3 Comparison of butterfly mean occupancy trends to trends from standardised sampling. Comparison of estimates of mean occupancy change from the occupancy-detection models developed in this study with estimates of mean occupancy change based on standardised sampling campaigns performed in the course of the biodiversity monitoring program of Switzerland BDM[2]. BDM data were not included in the occupancy-detection models for the comparison. The BDM only started in 2003, thus only occupancy changes after 2003 are included. For each species, change estimates between certain years are compared between the two models. These comparisons are made for different time spans between years, ranging from one year (comparing each year's mean occupancy with the following year's mean occupancy) up to 17 years (comparing first and last available year's occupancies). **a**, Proportion of comparisons that are in the correct quadrant (see panel b), i.e. for which both models estimate a positive change or both models estimate a negative change, for different time spans (1 to 17 years, on the x-axis). The different colours show these comparisons for different subsets, i.e. when all estimates are included, when only estimates which show at least significant changes in the BDM reference are included, when only estimate which show at least significant changes in the occupancy-detection models are included, or when only estimates which show significant changes in both models are included. **b**, Comparison of estimates of change between the two models for three different time spans (1, 9, 17 years). Each point shows a species and pair of years combination (change in mean of posterior distribution), and grey segments show 95% credible intervals. Colours indicate significance of the estimates, i.e. whether none of the estimate was significant, whether only the BDM reference was significant, whether only the occupancy-detection model was significant or whether both models were significant. The black line is the trend line.

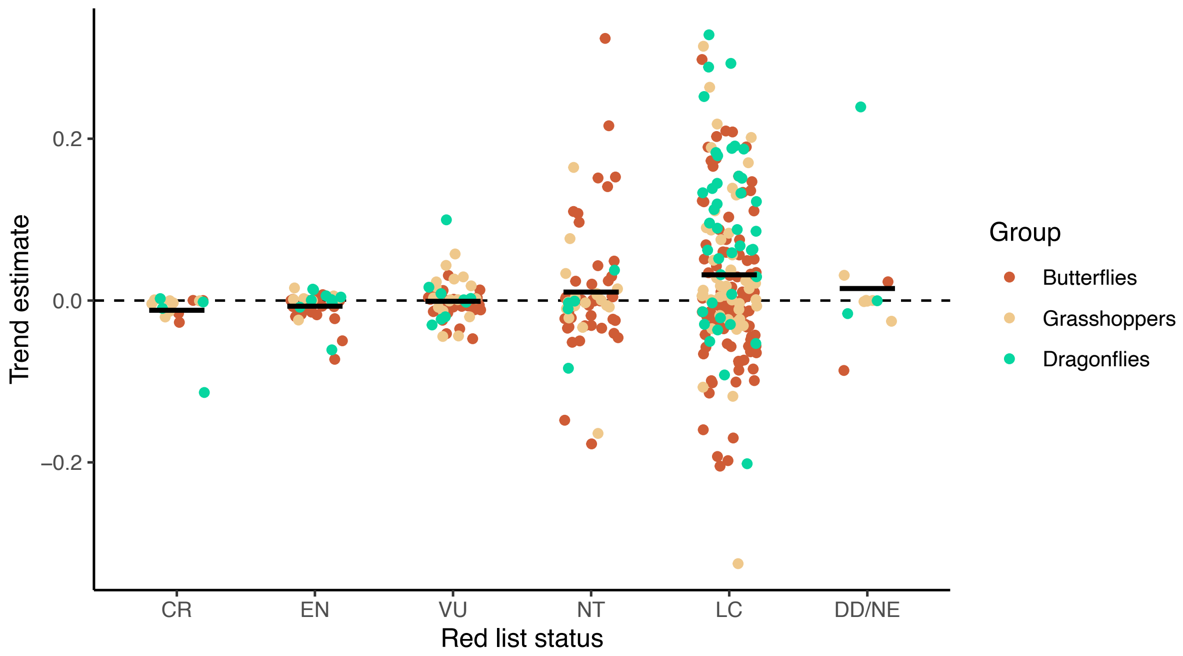

Fig. S4 Species trends by Red List status. 40-year species trends (point estimates from Fig. 2) shown along their Red List status based on group-specific Red Lists for Switzerland[3–5]. Point colours indicate insect group, horizontal segments show trend means. Increasing mean trends when moving from more to less threatened species are apparent. CR: Critically endangered; EN: Endangered; VU: Vulnerable, NT: Near threatened; LC: Least concern, DD/NE: Data deficient/Not evaluated.

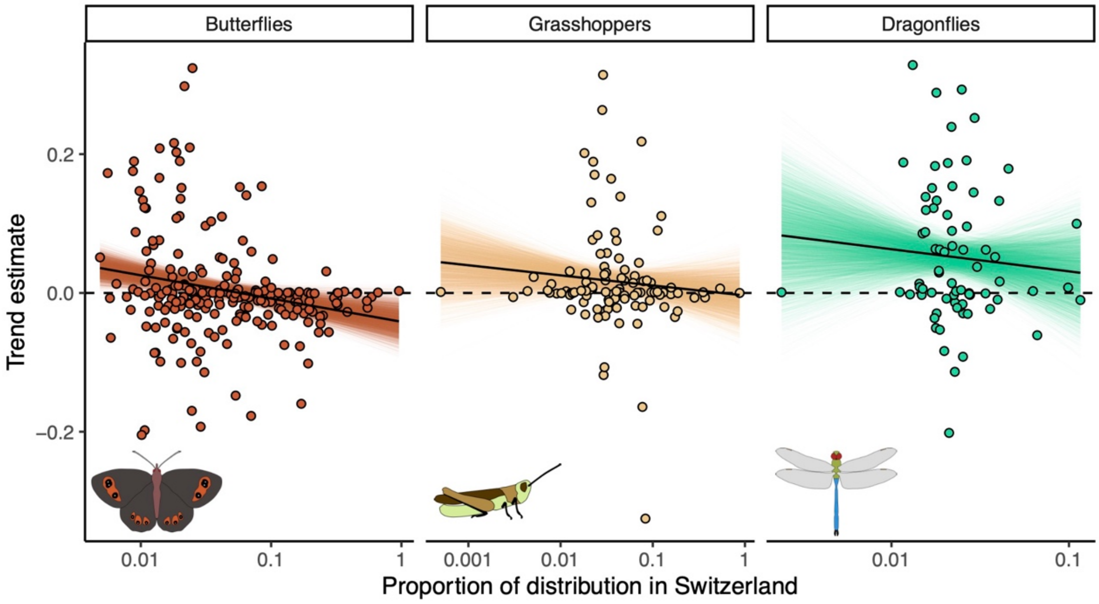

Fig. S5 Trend estimates against worldwide distributions. Species trend estimates across 40 years (1980–2020) from occupancy-detection models against the proportion of their worldwide distribution that lays in Switzerland, shown for the three groups separately. The worldwide distribution was inferred from GBIF data (https://doi.org/10.15468/dl.t6ha3h for butterflies, https://doi.org/10.15468/dl.reemkv for grasshoppers, https://doi.org/10.15468/dl.czbrmq for dragonflies) and aggregated at the ISEA10 grid[6] to partly correct for sampling bias. Lines show the posterior distribution of the slope and its mean from a linear model (proportion was log-transformed), the 95% credible interval is [-0.0233, -0.00540] for butterflies, [-0.0202, 0.00707] for grasshoppers and [-0.0566, 0.0327] for dragonflies ([-0.0227, -.00687] when all groups are analysed at once). Thus, species with larger proportions of their global distribution in Switzerland showed stronger decreases than species with smaller proportions. Species for which Switzerland harbours large parts of their available populations experienced strongest declines in the last 40 years. These mainly alpine, cold-adapted species seem to be more threatened with extinction[7–9], not only locally but also globally.

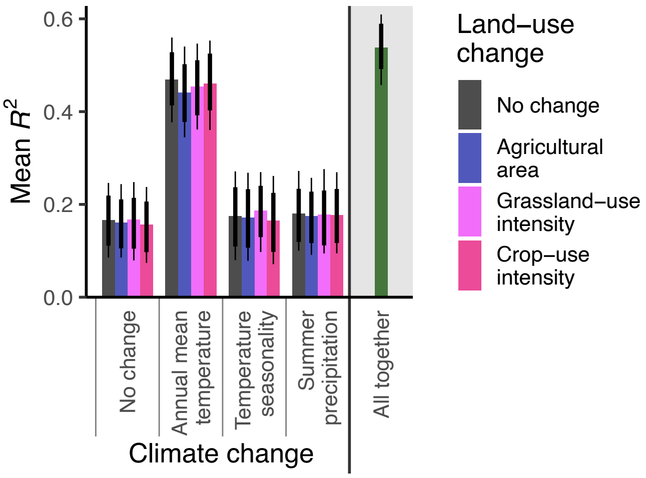

Fig. S6 Explained variance of long-term species trends for different climate and land-use scenarios based on the full set of species. Based on the results of the regression model including all species (Table S4), 40-year trends in mean occupancy (across whole of Switzerland) were predicted for all 390 species for scenarios of no climate and land-use change (i.e. change assumed to be zero across all zones and time intervals) and for different combinations of single measured trajectories of climate and land-use variables. The R^2 values (squared Pearson correlations) indicate how well the predictions align with the observed long-term species trends. The green bar on the right shows the match for the predictions with all climate and land-use variables following their measured trajectories (analogous to the R^2 of the model presented in Fig. 3a). Bars show means of the posterior distribution; vertical lines show 80%- and 95% credible intervals. Figure is the analogue of Fig. 4, but based on the full set of species.

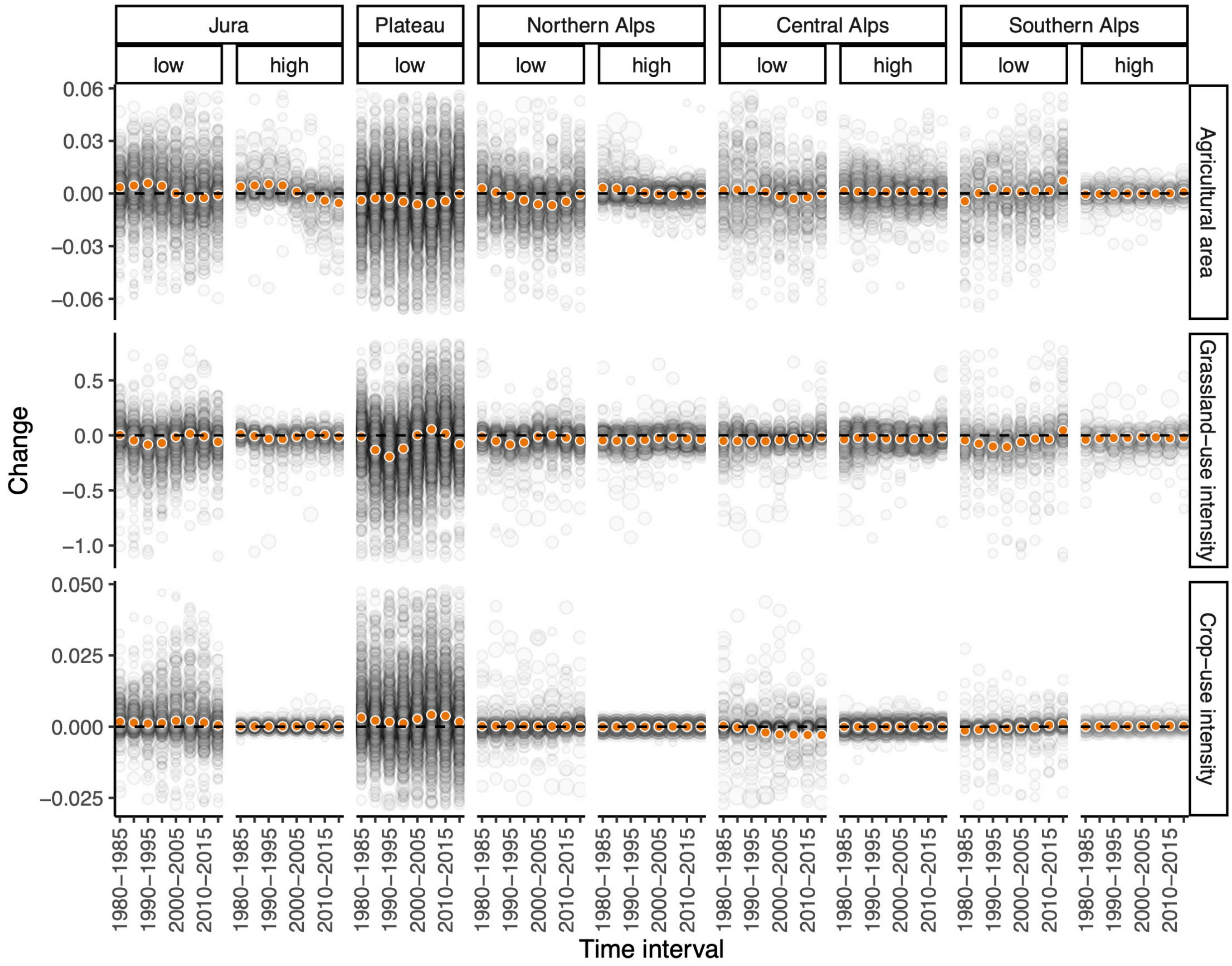

Fig. S7 Land-use change at small scale (municipality). Change of the three land-use variables (total agricultural area, grassland-use intensity, crop-use intensity) across consecutive 5-year intervals at the level of single municipalities, which are the level of recording of agricultural censuses and statistics. Estimates are sorted by the bioclimatic zones (municipalities overlapping several zones were split) and are based on the same generalised additive model approach used for regional estimates. Each grey point shows the change in a municipality in a 5-year time interval. Size of points relates to area of municipalities. Extreme outliers (upper and lower 1% of observations) were excluded to improve readability. Orange points show changes at regional scales (Fig. 1c), which were used in the main analyses of this study. Local land-use change includes larges magnitudes of change compared to regional land-use changes. At regional scales, large local changes tend to neutralise. Still, land-use change at municipality level follows regional change in many cases, indicated by synchronised patterns of change across the study area.

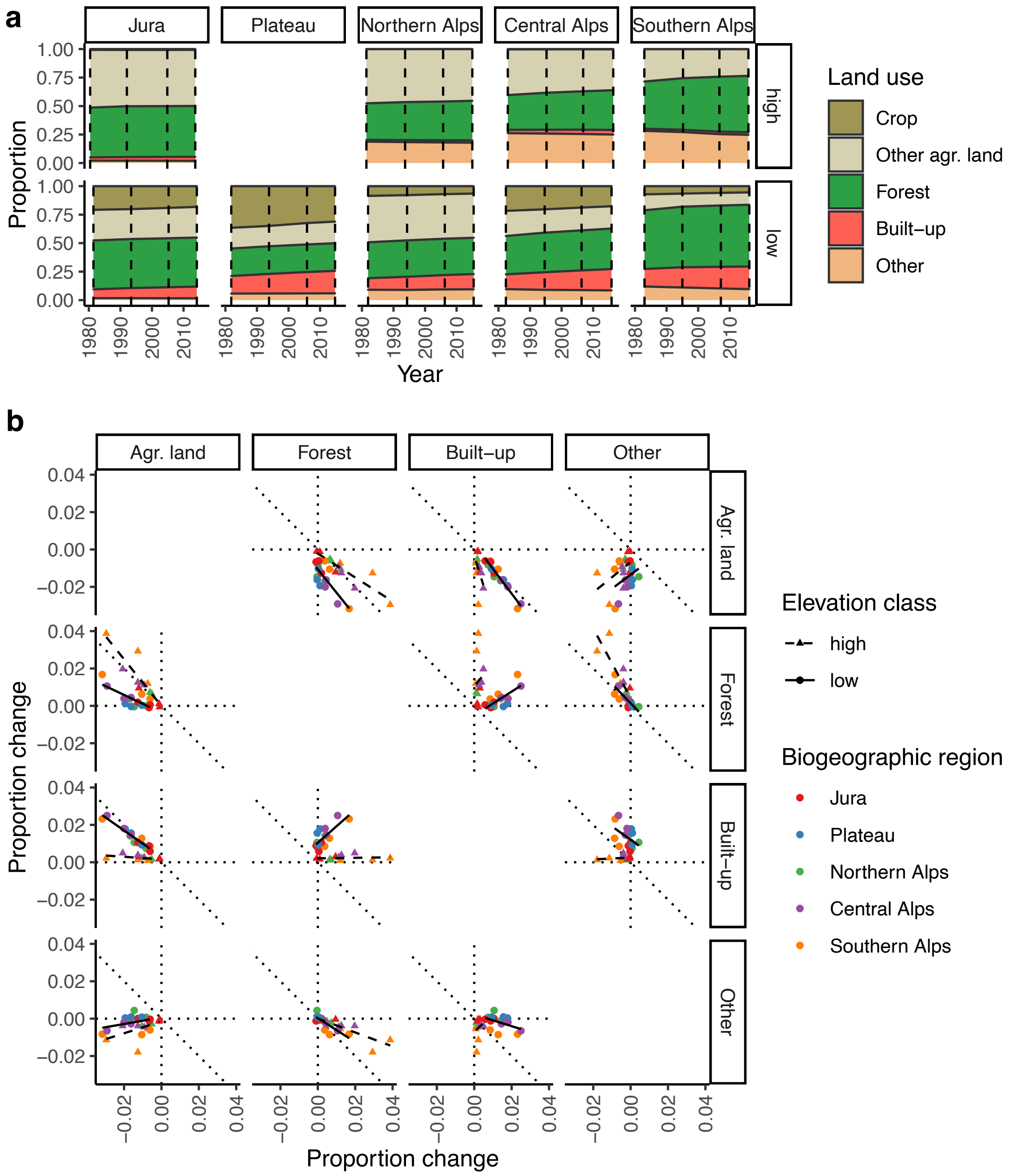

Fig. S8 Change of proportions of land-use types. a, Based on aerial photo interpretation for the whole of Switzerland at 4 time steps (dashed vertical lines) at the hectare resolution[10], proportions of different land-use types (crop, other agricultural land, forest, built-up, other) are shown. Upper panels show high elevation (> 1000 m asl), lower panels low elevation (< 1000 m asl). Data were restricted to squares for which species records were analysed. **b**, Change in proportions between consecutive measurement steps (panel (a)) set into relation between different land-use types. Colours indicate biogeographic regions, point shapes show the elevation class. Linear model slopes are shown separately for the two elevation classes. Dotted lines indicate no change (vertical, horizontal) and perfect match between change of two land-use types (slope -1). Change in agricultural land summarises change in the land-use types crop and other agricultural land from panel (a). Overarching patterns are a decrease of agricultural land (particularly crops) in low elevation due to spread of built-up areas and an increase of forests, particularly at high elevation, which coincides with a decrease of agricultural land.

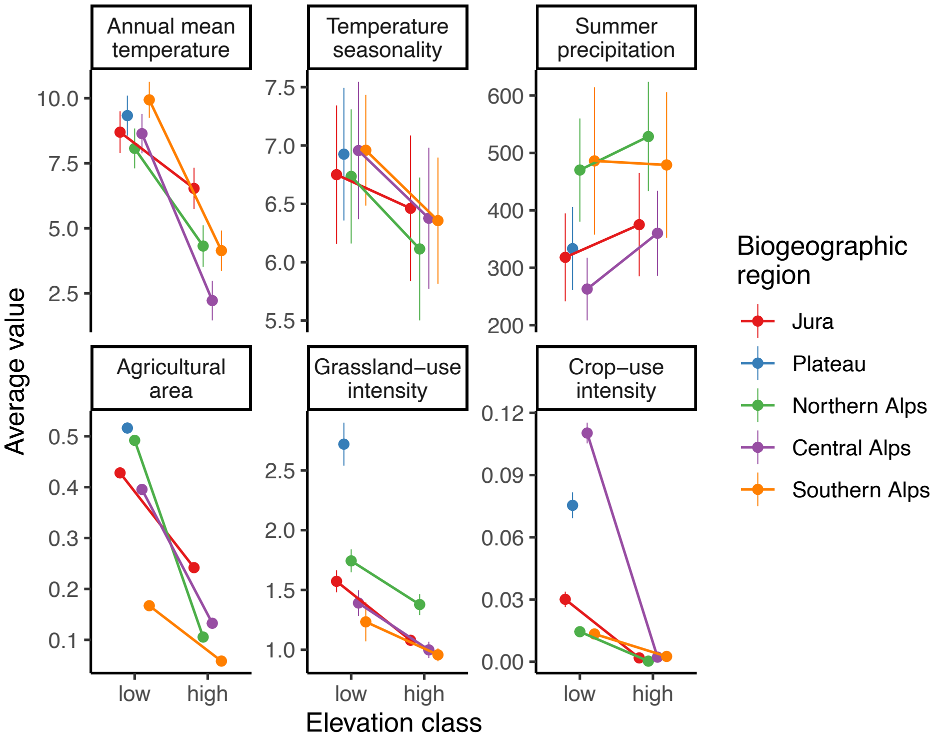

Fig. S9 Mean absolute values of the six climate and land-use change variables in the nine bioclimatic zones. Colours indicate the biogeographic regions, which are separated into the two elevation classes (above and below 1000 m asl.). Vertical segments indicate standard deviations with replication units being one year for climatic variables (top, $n = 41$ for each observation) and 5-year periods for land-use variables (bottom, $n = 8$ for each observation). Data were restricted to squares for which species records were analysed.

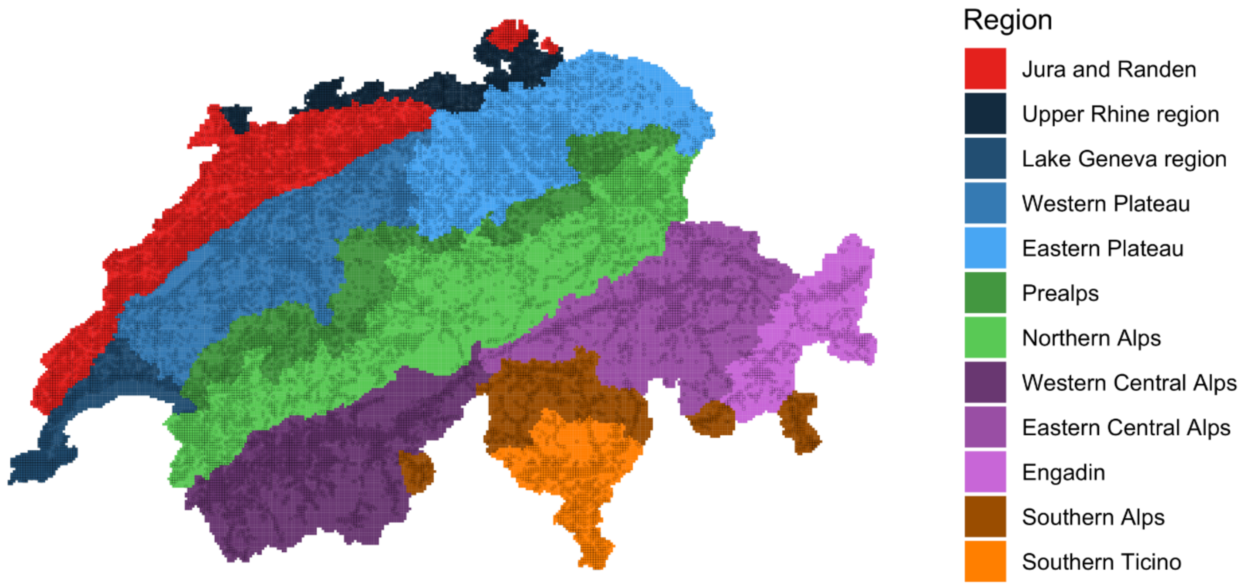

Fig. S10 The fine biogeographic regions of Switzerland. These twelve finer biogeographic regions[11] were used in the occupancy-detection models to account for spatial variability in occupancy. Squares for which records were analysed are shown in darker colour.

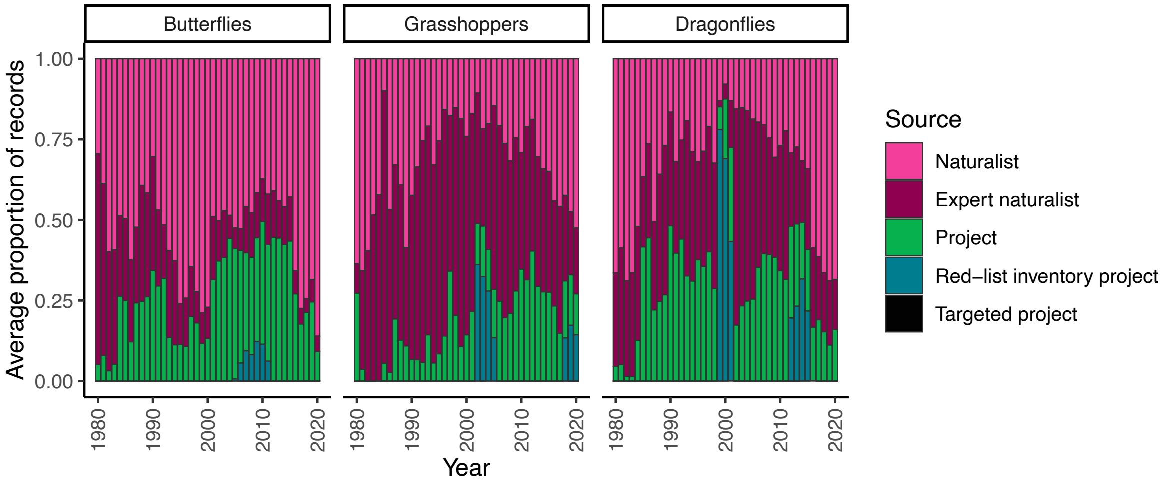

Fig. S11 Yearly average proportion of recordings originating from the different data sources, which were distinguished in occupancy-detection models. For each year (1980–2020), the average proportion of records originating from each of the data source categories across all study species is shown. Proportions are shown separately for each insect group. Records from targeted projects (i.e. projects aimed at a specific subset of species or even single species) were rare, such that they do not show in the figure.

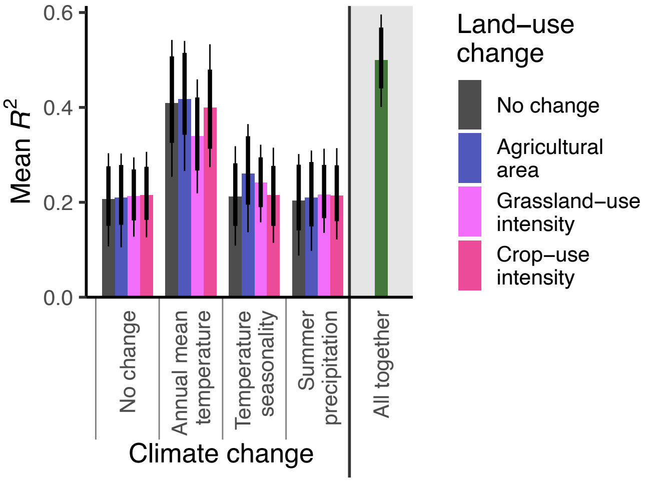

Fig. S12 Explained variance in predicted mean occupancy trends for different climate and land-use scenarios based on 10-year intervals. Based on the results of the full regression model only including species of agriculturally influenced habitats (second version), overall trends in mean occupancy (across the whole of Switzerland, 1980–2020) were predicted for all 276 species for scenarios of no climate and land-use changes and for different combinations of single measured trajectories of climate and land-use variables. The R^2 values (squared Pearson correlations) indicate how well the predictions align with the observed species trends. The green bar on the right shows the match for the predictions with all climate and land-use variables following their measured trajectories. Bars show the means of the posterior distribution, vertical lines show 80%- and 95%-credible intervals. Figure is the analogue of Fig. 4, but based on 10-year intervals instead of 5-year intervals.

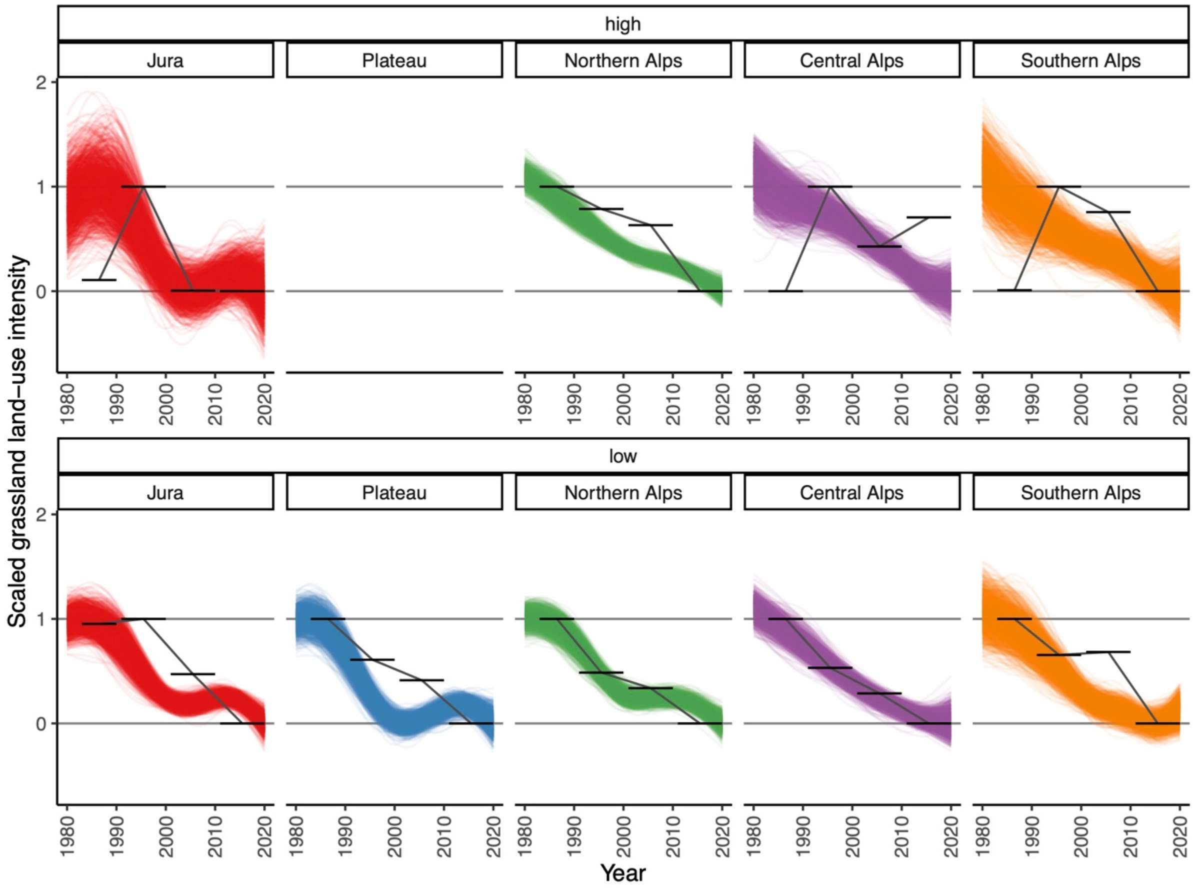

Fig. S13 Comparison of grassland-use intensity trends based on two different methods. Comparison of grassland-use intensity estimated from livestock units per grassland area (coloured lines; used in man analyses) and from satellite imagery (black segments) shown for the nine bioclimatic zones (five regions and two elevations). Coloured lines show single draws from the posterior distribution of additive models relating land-use intensity to the year. They have been scaled such that mean values range between 0 and 1 for the period 1983–2020 within each bioclimatic zone. Black segments show the mean estimated land-use intensity value for four time periods, across which data from satellite imagery were averaged (1983–1990; 1991–2000; 2001–2010; 2011–2020). Also, values were scaled to range between 0 and 1 within each bioclimatic zone. The grassland-use intensity estimated from satellite imagery is based on the method in ref.[12], which uses NDVI measurements. In comparison, the economic output variables were omitted to calibrate the model (as they were not retrospectively available). For the current state (2011–2020), model predictions were based on Sentinel-2 data, whereas for the three retrospective periods, Landsat-7&8 data were used.

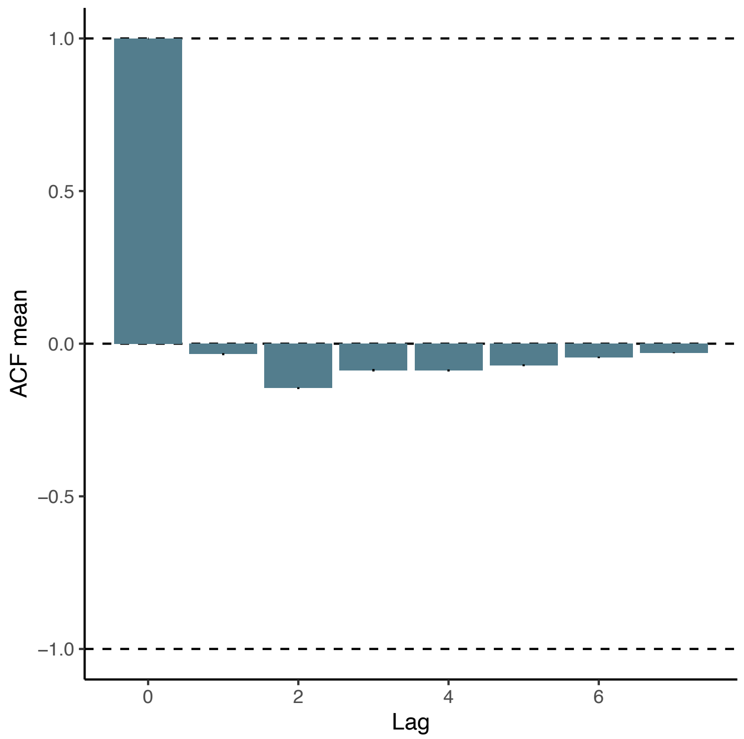

Fig. S14 Autocorrelation function (ACF) of trend model residuals. The ACF estimates temporal autocorrelation in the model residuals for different time lags. Based on the first model version in which parameters for change in agricultural area and grassland-use intensity were only included for species of agriculturally influenced habitats. Lag 1 corresponds to the autocorrelation of the residual of one 5-year interval to the residual of the next 5-year interval. Residuals were grouped by bioclimatic zone and species.

Supplementary References

1. Klaiber, J. *et al. Fauna Indicativa*. Swiss Federal Research Institute WSL, Birmensdorf, Switzerland, (2017). https://www.wsl.ch/publikationen/pdf/16419.pdf.
2. Koordinationsstelle BDM. *Biodiversitätsmonitoring Schweiz BDM. Beschreibung der Methoden und Indikatoren*. Bundesamt für Umwelt, Bern, Switzerland, (2004). https://www.bafu.admin.ch/bafu/de/home/themen/biodiversitaet/publikationen-studien/publikationen/biodiversitaetsmonitoring.html.
3. Monnerat, C., Thorens, P., Walter, P. & Gonseth, Y. *Rote Liste der Heuschrecken der Schweiz*. Bundesamt für Umwelt, Bern, Switzerland / Schweizer Zentrum für die Kartografie der Fauna, Neuenburg, Switzerland, (2007). https://www.bafu.admin.ch/bafu/de/home/themen/biodiversitaet/publikationen-studien/publikationen/rote-liste-heuschrecken.html.
4. Monnerat, C., Wildermuth, H. & Gonseth, Y. *Rote Liste der Libellen. Gefährdete Arten der Schweiz*. Bundesamt für Umwelt, Bern, Switzerland / Schweizer Zentrum für die Kartografie der Fauna, Neuenburg, Switzerland, (2021). https://www.bafu.admin.ch/bafu/de/home/themen/biodiversitaet/publikationen-studien/publikationen/rote-liste-libellen.html.
5. Wermeille, E., Chittaro, Y. & Gonseth, Y. *Rote Liste Tagfalter und Widderchen. Gefährdete Arten der Schweiz, Stand 2012*. Bundesamt für Umwelt, Bern, Switzerland / Schweizer Zentrum für die Kartografie der Fauna, Neuenburg, Switzerland, (2014). https://www.bafu.admin.ch/bafu/de/home/themen/biodiversitaet/publikationen-studien/publikationen/rote-liste-tagfalter-und-widderchen.html.
6. Carr, D., Kahn, R., Sahr, K. & Olsen, T. ISEA discrete global grids. *Stat. Comput. Stat. Graph. Newsl.* **8**, 31–39 (1997).

7. Bowler, D. E. *et al.* Winners and losers over 35 years of dragonfly and damselfly distributional change in Germany. *Divers. Distrib.* **27**, 1353–1366 (2021).

8. Steinbauer, M. J. *et al.* Accelerated increase in plant species richness on mountain summits is linked to warming. *Nature* **556**, 231–234 (2018).

9. Illich, I. & Zuna-Kratky, T. Population dynamics of an alpine grasshopper (Orthoptera) community over 30 years and the effects of climate warming and grazing. *J. Insect Conserv.* **26**, 435–451 (2022).

10. Bundesamt für Statistik. *Arealstatistik nach Nomenklatur 2004 – Bodennutzung (Land Use)*. (2015). https://www.bfs.admin.ch/bfs/de/home/statistiken/raum-umwelt/nomenklaturen/arealstatistik/nolu2004.html.

11. Bundesamt für Umwelt. *Die biogeografischen Regionen der Schweiz. 1. aktualisierte Auflage 2022.* Bundesamt für Umwelt, Bern, Switzerland, (2022). https://www.bafu.admin.ch/bafu/de/home/themen/landschaft/publikationen-studien/publikationen/die-biogeographischen-regionen-der-schweiz.html.

12. Meier, E. S., Indermaur, A., Ginzler, C. & Psomas, A. An effective way to map land-use intensity with a high spatial resolution based on habitat type and environmental data. *Remote Sens.* **12**, 969 (2020).
